# Second International Lygus Symposium: Asilomar Conference Grounds, Pacific Grove, California, April 15-19 2007

**DOI:** 10.1673/031.008.4901

**Published:** 2008-09-17

**Authors:** P. B. Goodell, Peter C. Ellsworth

**Affiliations:** ^1^ Kearney Agricultural Center University of California, Kearney, CA IPMpbg@uckac.edu; ^2^ Arizona Pest Management Center & Department of Entomology, Maricopa Agricultural Center, The University of Arizona, Maricopa, AZ cmckenzie@ushrl.ars.usda.gov

## Abstract

The Second International Lygus Symposium brought together 52 entomologists from six nations and 11 states representing universities, public agencies, and private entities to discuss the latest research on *Lygus* species and their relatives. Symposium topics included *Lygus* biology, behavior and ecology, IPM, insecticides and resistance, and biological control. Papers and posters dealt with *Lygus* as a pest of several crops, including cotton, strawberries, seed alfalfa, canola, dry beans, cucumbers, cereals, peaches, and new crops guayule and lesquerella. Intercrop movement of *Lygus*2008200820082008 species was another important topic of many presentations. In the capstone session, participants identified needs and priorities for ongoing *Lygus* research and education (available at http://ag.arizona.edu/apmc/Arid_SWPMC_RAMP.html). The conference was sponsored in part by FMC Corporation, the University of Arizona Arizona Pest Management Center, the University of California Statewide IPM Program, and a grant to Ellsworth et al. (CRIS# 0207436) from the USDA-CSREES, Risk Avoidance and Mitigation Program (RAMP).

Functional genetics implies a robust understanding of the characteristics of a gene, including where, when, and how it behaves in an organism. This level of investigation has been mostly limited to model organisms such as yeast or *Drosophila*. However, current technology and bioinformatics allow for studies on non-model organisms such as the tarnished plant bug, *Lygus lineolaris*. As a first step in identifying functional genes, a cDNA library was prepared from a laboratory-reared colony of *L. lineolaris* male nymphs. A small number of sequences were obtained and compared to known genes. Sequences that appeared to have known functions or close homologues have been targeted for further study. Three sequences appeared to encode polygalacturonase (PG) enzymes, and cDNAs were cloned in their entirety by Rapid Amplification of cDNA Ends (RACE). Other sequences of interest include actins, tubulins, ribosomal subunits, proteinases, and cuticle genes. To identify expression profiles for these genes, as related to developmental stage and sex of the insect, total RNA samples were collected from eggs at 3 and 8 days of development, 2nd instar nymphs, 5th instar nymphs (identifiable as either male or female) and adults, male and female. Equal quantities of total RNA were reverse transcribed and used for semiquantitative PCR. Several genes, including the PGs, were expressed with stage- and sex-specificity. Constitutively expressed genes were identified and will be useful as standards.

