# Supplementary material for: Second International Lygus Symposium: Asilomar Conference Grounds, Pacific Grove, California, April 15-19 2007
Source: J Insect Sci. 2008 Sep 17;8:49. doi: 10.1673/031.008.4901 (PMC3127420; doi:10.1673/031.008.4901)
Supplement: Related texts

# Research article: Landscape Modeling of Lygus hesperus Populations

**DOI:** 10.1673/031.008.4903

**Published:** 2008-09

**Authors:** Jay Bancroft, Peter Goodell

**Affiliations:** ^1^ USDA-ARS, Shafter, CA, USA; ^2^ Kearney Agricultural Center University of California, Kearney, CA, USA

*Lygus* bugs are a key pest in several of the 200 crops grown in the geographically distinct San Joaquin Valley. Spring weather patterns drive population build-up on host-plants. Habitat maps were created from survey sites in cropping regions of Kern County, CA. Parameters were calibrated from literature and dispersal experiments. Population simulations were used to predict abundance and movement among the mosaic of landcover types. The results provide estimates of pest pressure in target areas. General conclusions about risk from landscape configuration may be used in other contexts.

# Research article: Age specific fecundity of Lygus Hesperus in high, fluctuating temperatures

**DOI:** 10.1673/031.008.4904

**Published:** 2008-09

**Authors:** Jay Bancroft, Rikki Thompson, Maria Garcia, Jason Welch

**Affiliations:** USDA-ARS, Shafter, CA, USA

We have simulated hourly temperatures to examine the response of *Lygus hesperus* to hot summers in the San Joaquin Valley. Constant temperature of 33°C quickly killed *L. hesperus* and San Joaquin Valley temperatures routinely surpass this level. Average hourly temperatures were tested for the months May, July, and September. Age specific fecundity was measured using our standard bean/sunflower seed diet. Intrinsic rate of increase was greatest in the July temperature treatment, with average temperature of 26.3°C *Lygus hesperus* was readily able to tolerate several hours above 34°C, the high temperature in the July treatment. The results show typical summer heat is likely to cause only a small adverse affect on *Lygus* populations, which corroborates observed seasonal population dynamics.

# Research article: Intercept Traps for Monitoring Lygus Flux Between Fields

**DOI:** 10.1673/031.008.4905

**Published:** 2008-09

**Authors:** Jay Bancroft, Peter Goodell

**Affiliations:** ^1^ USDA-ARS, Shafter, CA, USA; ^2^ Kearney Agricultural Center University of California, Kearney, CA, USA

Window pain traps integrate over the time of deployment and are useful for monitoring the movement of insects. In previous designs, small glass or Plexiglas frames intercept insects in flight, causing them to fall into a collecting reservoir. We have modified this idea by increasing the size of the interception area in order to detect the movement of arthropods, including *Lygus hesperus*. They have been especially useful for measuring dispersal from alfalfa to cotton. This intercept trap is 1 × 2 meters and is easily constructed. The trap consists of aluminum T-bar with wooden slates which form the frame. Plastic pallet wrap is used as the clear “window”. Inexpensive plastic rain gutters provide the collecting reservoir when filled with antifreeze. This trap provided good evidence of *Lygus* movement from swathed alfalfa to cotton when it was placed between the two fields. Maximum movement peaked at 48 hours after cutting and dropped off rapidly after that. This tool provides relative numbers of movement and the direction of movement based on which side of the trap was struck and therefore, into which gutter the insect fell. We are now using sample transects in source and destination fields (along with mortality estimates) to calibrate the efficiency of the trap.

# Research article: Insecticide resistance and efficacy of novaluron and flonicamid for control of Lygus bugs in alfalfa seed

**DOI:** 10.1673/031.008.4906

**Published:** 2008-09

**Authors:** James Barbour

**Affiliations:** Parma Research and Extension Center, Department of Plant Soil and Entomological Sciences, University of Idaho, Parma, ID, USA

*Lygus* bugs, mostly *Lygus hesperus* Knight and *L. elisus* Van Duzee (Heteroptera: Miridae) are the most important insect pest of alfalfa seed in Idaho, commonly exceeding established economic thresholds and commonly require three or more pesticide applications each growing season for their control. Because alfalfa seed yields are highly dependent on the number and activity of bees used for pollination, management strategies that limit negative impacts on pollinators are key elements in integrated management programs for *Lygus* bugs in alfalfa seed. *Lygus* bugs in alfalfa seed are primarily managed by application of broad spectrum organophosphate, carbamate and pyrethroid insecticides. Although a number of compounds are currently available, the usefulness of these compounds is limited by a number of factors including their toxicity to pollinators, insecticide resistance management, and the presence of other pests and presence of predators and parasitoids of *Lygus* bugs and other pests. Experiments were conducted in 2006 to determine if two new, low risk compounds, novaluron and flonicamind, could provide effective control of *Lygus* when applied pre-bloom (prior to bee release). Numbers of *Lygus* were very high during the study with peak numbers exceeding 70 per sweep on untreated plots. Although most treatments reduced numbers of *Lygus* nymphs below levels found in untreated plots, none reduced numbers to threshold levels. Numbers of nymphs were lower on plots treated with flonicamid or lambda cyhalothrin, but remained above threshold on all plots. Treatments combining novaluron with lambda cyhalothrin appeared to give better control than novaluron alone. Alfalfa seed yields which were also higher in plots treated with flonicamid, lambda cyhalothrin and lambda cyhalothrin plus novaluron than in untreated plots.

# Research article: Endigo™ a new, enhanced product with two modes of action to protect cotton against key mid-to late-season pests

**DOI:** 10.1673/031.008.4907

**Published:** 2008-09

**Authors:** David Belles, Catherine Savinelli

**Affiliations:** ^1^ Syngenta Crop Protection Inc., Chandler, AZ, USA; ^2^ Syngenta Crop Protection Inc., Greensboro, NC, USA

Endigo™ is a new, enhanced product with two modes of action to protect cotton against key mid- to late-season insect pests, including *Lygus lineolaris*. Endigo combines lambda-cyhalothrin an IRAC group 3 pyrethroid with thiamethoxam a group 4A neonicotinoid. This combination controls most key mid-to late season cotton insect pests by providing quick knockdown and long residual control thereby helping to achieve maximum cotton yield and quality. Endigo has an ideal use window from full bloom to cutout and is a convenient way to clean up fields at the end of the season. Endigo may be applied at 3.5 to 5.5 fl. oz/A as required by scouting at 5 to 10 day intervals. The maximum application rate per season is 13.5 fl. oz/A with a minimum of at least 5 days between application. Endigo may be applied by ground or air at application volumes of 10 and 3 gallons per acre, respectively. Endigo has a re-entry period of 24 hours following application and a pre-harvest interval of 21 days. Livestock should not be grazed in treated areas. *Lygus lineolaris* control in Mississippi with 4 and 5 oz/A of Endigo was 75 and 81%, respectively, compared to 71% with Bidrin at 4 oz/A plus Capture at 2.6 oz/A. In Tennessee, *Lygus lineolaris* control with 4 and 5 oz/A of Endigo was 96 and 98%, respectively, compared to 83% with Bidrin at 4 oz/A plus Capture at 2.6 oz/A. The use of Endigo should conform to sound resistance management strategies established for the crop and use area. At a minimum, avoid using a block or more than three consecutive applications of Endigo and/or other Group 4A insecticides. Following a block of group 4A insecticides, rotate to a block of products with a different mode of action. Incorporate IPM techniques into an insect control program and monitor insect populations for field efficacy. Endigo is not currently registered for sale or use in the United States.

# Research article: Flonicamid mode of action studies

**DOI:** 10.1673/031.008.4908

**Published:** 2008-09

**Authors:** Bruce Black, Joe Argentine, Rathnam Chaguturu, Ratna Dargar, Leo Dungan, Russ Eldridge, Lyle Kinne, Russ Peters, Amy Wrzesinski, Debbie Yuhas

**Affiliations:** FMC Corporation, Agricultural Products Group, Princeton, New Jersey, USA

Flonicamid offers a new opportunity to control insects through what appears to be a unique mode of action. Results from a battery of mode of action assays differentiate flonicamid from the most common commercial insecticides used in US crop protection today. Assays performed to date include the inhibition studies of acetylcholinesterase, nicotinic and muscarinic acetylcholine receptors, respiration (including Complex I, II, III, and IV inhibitors, phosphorylation inhibitors, uncouplers of oxidative phosphorylation), GABA-receptor (*in vivo* and *in vitro*), glutamate-receptor (agonist and antagonist), octopamine receptor agonist, nitric oxide synthase, nitric oxide receptor agonist, Na-channel, L-type Ca-channel, ryanodine Ca-channel, and calcium ATPase (body wall contraction assay). In addition, the rapid onset of toxicity (one hour) is inconsistent with chitin synthesis inhibition, juvenile hormone and ecdysone agonist activity. For these reasons, flonicamid does not appear to be an insect growth regulator. In all of the above assays, flonicamid does not act on these targets as do reference commercial standards. Collectively, these modes of action cover the majority (and more) of known insecticide modes of action. Lack of response by flonicamid in these assays compared to known standards suggests a novel mode of action that is differentiated from other insecticides commonly used in American agriculture. In a companion article, Hayashi et al. (2006) presents evidence that flonicamid targets the insect potassium A-type channel. Hence, suggesting a novel mode of action for flonicamid.

# Research article: Feeding disruption in Myzus persicae by a new insecticide, flonicamid

**DOI:** 10.1673/031.008.4909

**Published:** 2008-09

**Authors:** C Bruce Black, Bill Gravelle

**Affiliations:** FMC Corporation, Princeton, NJ, USA

An important feature of a piercing/sucking insecticide is its ability to rapidly inhibit feeding. An assay was developed to measure feeding cessation in aphids. 14C-Inulin is a non-metabolizable sugar that can be co-administered along with an insecticide in the aphid diet using feeding chambers. Honeydew was quantitatively collected from the lids of the feeding chambers and quantified. Aphids were introduced into chambers and allowed 36 hours to acclimate and begin feeding. At zero time, the diet was changed to a diet containing 14C-Inulin as well as the test compound administered at 100X the calculated LD50. The experiment was performed two ways: Cumulative honeydew was monitored (i.e. from time=0 to time point) and as rates/hour collected as timed intervals collected during the study. Both approaches gave essentially identical results. Feeding behavior stops immediately (extrapolated to t=0) for flonicamid upon insecticide exposure. On the other hand, mortality did commence until 35 hours of exposure (t1/2=40 hours). Honeydew recovered from treated aphids was evaluated for oligosaccharide composition. The ability to form complex polysaccharides was not affected during intoxication.

# Research article: Evaluation of color traps for monitoring Lygus spp.: design, placement, height, time of day, and non-target effects

**DOI:** 10.1673/031.008.4910

**Published:** 2008-09

**Authors:** L Jacquelyn Blackmer, A John Byers, Cesar Rodriguez-Saona

**Affiliations:** ^1^ US ALARC-ARS-USDA, 21881 N. Cardon Lane, Maricopa, AZ, USA; ^2^ PE Marucci Blueberry and Cranberry Center, 125a Lake Oswego Rd., Chatsworth, NJ, USA

*Lygus hesperus* and *L. lineolaris* are two of the most economically important plant bugs in North America. Here we present results from field trials that evaluated effective trap characteristics for maximizing *Lygus spp*. and other herbivorous insect capture, while minimizing beneficial insect capture. The response of *Lygus* bugs, several other key herbivore species and predators to hue (white, clear, black, yellow, orange, blue, purple, green and red) and value (black, white and two neutral grays) was examined in alfalfa over three seasons using traps coated with Pestick adhesive. *Lygus spp*. exhibited a broad response to trap hue, but showed no response to trap value. Additionally, we showed that time of day, trap height, and trap placement influenced the number of *Lygus spp*. captured. More *Lygus spp*. were trapped from late afternoon to dusk compared to all other times of the day and night, and more males than females were captured on sticky traps even though the sexes were at parity in field sweep net samples. In the alfalfa setting, male *Lygus* were more likely than females to be captured on traps placed 20 cm above the ground; traps placed 50 and 100 cm above the ground caught similar numbers of males and females. The highest number of plant bugs was captured when traps were placed in a cleared area between two alfalfa fields; lower numbers were captured on traps at the edge and in the center of the field. All other herbivores exhibited distinct preferences to trap hue and in some cases, trap value. Predators were rarely trapped, but did exhibit preferences to trap color (i.e., hue and value) characteristics. The potential of using sticky traps with specific hue and value characteristics to effectively monitor *Lygus spp*. is discussed.

# Research article: Implementing biological control of Lygus lineolaris in strawberries in Ontario, Canada

**DOI:** 10.1673/031.008.4911

**Published:** 2008-09

**Authors:** Bruce A. Broadbent, G. Peter Mason, Tim Haye

**Affiliations:** ^1^ Agriculture and Agri-Food Canada, London, Ontario, Canada; ^2^ Agriculture and Agri-Food Canada, Ottawa, Ontario, Canada; ^3^ CABI Europe Switzerland, Delemont, Switzerland

The populations of *Lygus lineolaris* adults and nymphs and levels of native larval parasitism were measured weekly at 6 strawberry farms in Ontario during the summer of 2006, as part of the first year of a project to introduce *Peristenus digoneutis* to the management of *Lygus* in strawberries. At farms with pesticide inputs, *Lygus* levels were very low on strawberries, but were detected at higher levels on adjacent alfalfa fields and weedy borders. Larval parasitism was found to be between 5–20% at these latter sites, with occasional levels of 30–40% parasitism on some collection dates. In the summers of 2007 and 2008 we intend to intensively sample 3 non-sprayed strawberry farms having both alfalfa trap crops and releases of *Peristenus digoneutis* at peak N2 levels of *Lygus* in May and July. Biological control agents are expected to contribute to the long-term reduction of *Lygus* populations in the region and are not seen as a short-term solution.

# Research article: Egg parasitoids of Lygus bugs in southern Alberta

**DOI:** 10.1673/031.008.4912

**Published:** 2008-09

**Authors:** Héctor Cárcamo, John Huber, Tracy Larson, Rob Bourchier

**Affiliations:** ^1^ Lethbridge Research Centre, Agriculture and Agri-Food Canada, Lethbridge, Alberta, Canada; ^2^ Canadian Forest Service, Ottawa, Ontario, Canada

Biological control is a promising alternative for the management of *Lygus* bugs, an important pest of seed alfalfa and oilseed crops in western Canada. Recently, the native nymphal parasitoids have been described but very little is known about the egg parasitoids attacking *Lygus* in this region. The objectives of this study were (1) to determine which egg parasitoids attack *Lygus* bugs in Southern Alberta in both cultivated and non-cultivated habitats, during peak *Lygus* oviposition periods and (2) quantify rates of parasitism of the *Lygus* eggs to improve understanding of *Lygus* mortality factors. In 2005 and 2006, egg parasitism was examined using a combination of sentinel *Lygus elisus* or *L. keltoni* eggs, laid in the lab on sprouted potato tubers and canola, and sentinel *Lygus* eggs laid on caged plants in the field. We also exposed sentinel egg cards with Mediterranean flour moth eggs (*Ephestia kuehniella*) in the same sites, as potential alternative host for egg parasitoids. To supplement sentinel eggs, *Lygus* host plants (flixweed [*Descurainia sophia*], lamb's quarters [*Chenopodium album*], stinkweed [*Thlaspi arvense*], mustard [*Sisymbrium altissimum*]) and also potential host-plants (absinth [*Artemisia absinthium*], buffalo bean [*Thermopsis rhombifolia*], yarrow [*Achillea sp*.]) were surveyed for naturally occurring *Lygus* eggs. Sentinel eggs were exposed for between 2–3 days in the field. After field exposure sentinel and wild plants were collected and placed in cardboard emergence boxes. All emerging insects were collected in 70% EtoH for identification. *Lygus* did not oviposit on buffalo bean, yarrow or absinth. Sentinel egg cards with flour moth eggs and sprouted potato tubers did not produce any egg parasitoids indicating that these may not have been recognized as a host. Three parasitoids, *Anaphes iole, Telenomus sp*. and *Polynema sp*. were identified as putative *Lygus* egg parasitoids. Most egg parasitoids emerged from lamb's-quarter plants (N=11 *Anaphes iole*, N=3 *Telenomus sp*.), followed by mustard (N=7 *Anaphes iole*, N=2 *Polynema sp*.), flixweed (N=4 *Anaphese iole*, N= 3 *Polynema sp*.) and finally alfalfa (N=2 *Anaphes iole*, N=2 *Polynema sp*.). Overall numbers of parasitoids were very low and similar in the agricultural and non-agricultural sites (N =10, vs 12, respectively). Other insects (aphids, leafhoppers other plant bugs), managed to “contaminate” the field-caged plants at high numbers in some sites, preventing definitive associations between egg parasitoid and *Lygus* hosts; nevertheless, total egg parasitism rates, estimated from total nymphal emergence, were very low (<5%) suggesting that egg parasitoids do not cause significant mortality of *Lygus* bugs.

# Research article: Parasitism of Lygus bugs by native Peristenus species in southern Alberta, Canada.

**DOI:** 10.1673/031.008.4913

**Published:** 2008-09

**Authors:** Héctor Cárcamo, Henri Goulet, Carolyn Herle, Jennifer Otani

**Affiliations:** ^1^ Lethbridge Research Centre, Agriculture and Agri-Food Canada, Lethbridge, Alberta, Canada; ^2^ ECORC, Agriculture and Agri-Food Canada, Ottawa, Ontario, Canada; ^3^ Beaverlodge Research Farm, Agriculture and Agri-Food Canada, Beaverlodge, Alberta, Canada

Managing *Lygus* bugs in field crops of the Canadian prairies relies on insecticides as no alternatives are available. For example in 1998 over one million acres of canola were sprayed to reduce yield losses from an outbreak in Alberta. Seed alfalfa fields are prophylactically sprayed for *Lygus* and alfalfa plant bugs before releasing leafcutter bees. Encouraging results with biological control of *Lygus* bugs in northeast U.S.A. using introduced *Peristenus* wasps, have sparked interest in this group in Canada. The objective of this southern Alberta study were to (i) determine the suite of *Peristenus* species that attack *Lygus* bugs in and around crops, (ii) obtain baseline data on the levels of parasitism caused by these parasitoids, and (iii) document the basic seasonal distribution of the parasitoid species. Three *Peristenus* species were reared from *Lygus* nymphs: *P. carcamoi, P. broadbenti* and *P. braunae* with a putative fourth species to be confirmed. *Peristenus braunae* is rare in the first generation of *Lygus* in southern Alberta although it is commonly caught in sticky cards in grassy field margins in early May and may be more common in *Adelphocoris spp*. or other Miridae. By far the dominant species on the first *Lygus* generation was *P. carcamoi* which was collected from early June into late July suggesting that occasionally it attacks the second generation at low levels. *Peristenus broadbenti* was collected from mid-July into early August from the second generation nymphs. Total development times for each species were 350 and 352 days for *P. carcamoi* for males and females, respectively and 362 for both sexes of *P. broadbenti*. The number of days from adults to emergence from pupae after transferring them to 22 deg C from overwintering storage, were 4–7 for *P. carcamoi* but over 24 for *P. broadbenti*. Preliminary analysis of a no-choice laboratory oviposition study on *L. keltoni* (common host) and *L. lineolaris* (rare in the area) suggested that both *Peristenus* species have no preference for either *Lygus* bug host. Rates of parasitism from nymphal dissections ranged from zero up to around 60 % at some sites on some collection dates. Ongoing studies are focusing on quantifying the temporal dynamics of parasitism rates in alfalfa and canola grown adjacently to determine the potential for native *Peristenus* to reduce *Lygus* bugs from reaching pest status in crops.

# Research article: Area-wide pest management of Lygus hesperus

**DOI:** 10.1673/031.008.4914

**Published:** 2008-09

**Authors:** Yves Carrière, C Peter Ellsworth, Pierre Dutilleul, Christa Ellers-Kirk, Virginia Barkley, Larry Antilla

**Affiliations:** ^1^ Department of Entomology, The University of Arizona, Tucson AZ, USA; ^2^ Department of Plant Science, McGill University, Macdonald Campus, Ste-Anne-de-Bellevue, Québec, Canada; ^3^ Arizona Cotton Research and Protection Council, Phoenix, AZ, USA

Understanding the effect of cropping patterns on population dynamics, dispersal, and habitat selection of insect pests has been an unresolved challenge. We studied the western tarnished plant bug, *Lygus hesperus* (Knight) (Heteroptera: Miridae), in cotton during early summer in central Arizona. We used a general approach based on Global Positioning System (GPS) and Geographic Information System (GIS) technologies combined with spatial statistics, to assess the maximum distance at which forage and seed alfalfa, fallow fields with weeds, and cotton affect *L. hesperus* population density. Using a set of 50 cotton fields as focal fields, we found that forage and seed alfalfa as well as weeds acted as *L*. hesperus sources for these cotton fields. The source effect did not extend beyond 375, 500, and 1500 m for forage alfalfa, weeds, and seed alfalfa, respectively. Conversely, cotton fields acted as *L. hesperus* sinks, but this effect did not extend further than 750 m from the focal cotton fields. These findings suggest that specific spatial arrangements of these field types could reduce *L. hesperus* damage to cotton.

# Research article: Lygus hesperus polygalacturonase characterization and role in plant damage

**DOI:** 10.1673/031.008.4915

**Published:** 2008-09

**Authors:** Celorio-Mancera, Maria de la Paz Carl L. Greve, Hamid Ahmadi, Larry Teuber, A Kenneth Shackel, L Margaret Allen, M John Labavitch

**Affiliations:** ^1^ Department of Plant Sciences, University of California, Davis, CA, USA; ^2^ USDA ARS Biological Control of Pests Research Unit, Stoneville, MS, USA

The amino terminus, of a *Lygus hesperus* salivary gland protein revealing polygalacturonase (PG) activity in an SDS-PAGE activity gel assay, has been sequenced via Edman degradation. The N-terminal amino acid sequence shares homology with the predicted amino acid sequence for putative *L. lineolaris* PGs identified in an EST database. The molecular weight of the *L. hesperus* PG is about 35.5 KDa, similar to molecular weights predicted for the *L. lineolaris* PG sequences in the database. In addition, the in silico digestion of the putative *L. lineolaris* PGs yielded peptide sequences similar to those identified by liquid chromatography-mass spectrometry-mass spectrometry peptide fingerprinting of the *L. hesperus* PG. *L. hesperus* peptides represent 51% coverage of peptides predicted for *L. lineolaris* PG 1, 48% coverage of *L. lineolaris* PG 2, and 26% of *L. lineolaris* PG 3. Insects of the species *L. hesperus*, reared on artificial diet, were moved to a “collection diet” for recovery of saliva. Extracts of the fed-on collection diet showed PG activity in a semi-quantitative enzyme activity assay while the control (not fed-on collection diet) yielded no activity. Proteins from the fed-on and control collection diets were subjected to peptide fingerprinting and compared with the peptides predicted by the putative *L. lineolaris* PG sequences. PG peptides shared by *L. hesperus* and *L. lineolaris* were detected in the protein sample from fed-on collection diet. Our previous research has indicated that the microinjection of a partially purified PG protein, from a crude *L. hesperus* head-pronotum protein extract, into developing alfalfa florets, resulted in plant injuries similar to those caused by *Lygus* feeding. Moreover, while microinjection of an enzymatically active *Aspergillus niger* PG II recombinant protein produced damage symptoms (67% of the florets injected stopped in their development, were discolored, and/or abscised), the microinjection of an inactive, mutant *A. niger* PGII caused no damage (0% of the florets injected were symptomatic). This demonstrated that PG activity was required to cause floret damage.

# Research article: Polygalacturonase isozymes in Lygus hesperus salivary glands

**DOI:** 10.1673/031.008.4916

**Published:** 2008-09

**Authors:** Celorio-Mancera, Maria de la Paz, L Margaret Allen, Carl L. Greve, M John Labavitch

**Affiliations:** ^1^ Department of Plant Sciences, University of California, Davis, CA, USA; ^2^ USDA ARS Biological Control of Pests Research Unit, Stoneville, MS, USA

The feeding strategy of mirids has been referred to as “lacerate or macerate and flush feeding” which supports high rates of food intake. In other words, plant bugs digest the plant tissue extra-orally, producing a liquefied brew rich in simple nutrient molecules. The insect's salivary polygalacturonase (PG), amylase and protease presumably facilitate this feeding strategy. Some authors have suggested that hemipteran phytopathogenicity might depend principally on the proteins secreted from the salivary glands. Fungal PG has been considered an important virulence factor in the development of plant diseases, degrading the pectin component of the cell wall and facilitating pathogen ingress and colonization of host tissues. A plant's pathogen defenses include a role for the interaction of the plant's PG-inhibiting proteins (PGIPs) with pathogen PGs. Our previous research has provided evidence for cotton and alfalfa proteins that inhibit *Lygus hesperus* PG. Therefore, our immediate goal is the purification of the interacting proteins. Protein separation techniques yielded the partial purification of a number of *Lygus* PG isozymes based on their biochemical characteristics such as glycosylation and charge. The PG isozymes were isolated from salivary glands of wild insects collected at alfalfa field sites. Products of the enzymatic reaction catalyzed by the different partially purified isozymes were analyzed by high performance liquid chromatography (HPLC). Although further biochemical characterization of the isolated PGs is needed, the HPLC data suggest the presence of at least one endoacting PG and one exo-PG. Partial nucleotide sequences with high homology to *L. lineolaris* PGs have been obtained from *L. hesperus* salivary gland genomic DNA and cDNA, providing evidence for the presence of more than one pg gene, consistent with recent reports describing a PG gene family in *L. lineolaris*.

# Research article: Impact of field margin vegetation management of tarnished plant bug, Lygus lineolaris (Palisot de Beauvois) infestations in cotton, Gossypium hirstum (L.)

**DOI:** 10.1673/031.008.4917

**Published:** 2008-09

**Authors:** D.R. Cook, E Burris, B.R. Leonard, R. D. Burns

**Affiliations:** ^1^ Northeast Research Station, LSU AgCenter, St. Joseph, LA, USA; ^2^ Macon Ridge Research Station, LSU AgCenter, Winnsboro, LA, USA; ^3^ North Mississippi Research and Extension Center, Mississippi State University, Verona, MS, USA (Current Address)

A large-scale field study evaluated the effects of native winter-spring host plant management on tarnished plant bug, *Lygus lineolaris* (Palisot de Beauvois), infestations in cotton, *Gossypium hirsutum* (L.), in Tensas Parish, LA during 2004, 2005, and 2006. The treatments consisted of a herbicide-sprayed and a non-sprayed area on commercial farms located near Newellton and Waterproof, LA during each year. During 2004, both areas were ca. 611 ha. During 2005, the herbicide-treated area was ca. 1317 ha and the non-treated area was ca. 2094 ha. During 2006 the herbicide-treated area was 2094 ha and the non-treated area was 1317 ha. Each area (treated and non-treated) was divided into four quadrants. During January, 100 sample locations were established at each site (25 sites per quadrant). These locations were sampled with a 38.1 cm diameter sweep net at least bi-weekly during the period, February to June to estimate densities of tarnished plant bug, *Lygus lineolaris* (Palisot de Beauvois), adults and nymphs. During late February to early March, a herbicide combination (2, 4-D, mecoprop, and dicamba [Strike 3, Agriliance, LLC, St. Paul, MN]) was applied to the field margins in the herbicide-treated areas to destroy all broadleaf plants. These herbicide applications were made ca. three to five weeks prior to the associated fields being planted to cotton. Within the herbicide-treated and non-treated areas on each farm, cotton fields were randomly selected within each quadrant were sampled with a sweep net at least bi-weekly and the numbers of tarnished plant bug adults and nymphs were recorded from May until August. Tarnished plant bug numbers on native vegetation were similar at each site prior to herbicide application in all years. Following herbicide applications to field margins, numbers of tarnished plant bug adults and nymphs at the treated sites on native vegetation declined and remained low until the end of the sampling period during 2004 and 2005. During 2006, tarnished plant bug numbers were higher in the herbicide-treated areas compared to the non-treated area from early March until mid-April, after which tarnished plant bug numbers in the non-treated areas increased to levels above those in the herbicide treated area. During 2004, tarnished plant bugs were lower in cotton fields within the herbicide-treated areas compared to that in the non-treated areas during June and early July. During late July and early August, numbers of tarnished plant bug adults in cotton were similar between herbicide-treated and non-treated areas. Numbers of tarnished plant bug nymphs were greater than 2--fold higher in cotton fields at the non-treated site compared to fields in the herbicide-treated areas. During 2005. tarnished plant bug adults within the two sites were generally similar during mid-to-late June. Numbers of tarnished plant bug adults were ca. 2.6-fold higher in cotton fields in the non-treated areas compared to fields at the herbicide-treated areas during early July. During mid-to-late July, tarnished plant bug adults were higher in cotton fields within the herbicide-treated site compared to that in the non-treated areas. Densities of tarnished plant bug nymphs were 1.1-fold to 2.2-fold lower in cotton fields in the herbicide-treated areas compared to fields in the non-treated areas from mid June to late July. During 2006, tarnished plant bug adults and nymphs were higher in cotton fields in the herbicide-treated areas from late June until late July. Tarnished plant bug adults ranged from 1.2-fold to 1.9-fold higher in fields within the herbicide-treated areas compared to fields in the non-treated areas. Tarnished plant bug nymphs ranged from 1.0-fold to 2.8-fold higher in fields within the treated areas compared to fields in the non-treated areas. The sampling information for cotton fields within the two areas during 2006 were markedly different compared to the results collected during 2004 and 2005. The broad host range of tarnished plant bug includes many plants that occur along field margins and in non-crop area that may be adjacent to cotton fields. In the Mid-South, non-crop wooded areas and property enrolled in federal conservation programs occurred in close proximity to cotton fields. During all three years, these types of lands were present at both locations. Many of the available host plants that occurred in these non-crop areas could not be managed with the herbicide strategy evaluated in this study. During 2004, these types of lands that could not be treated with herbicides represented 8% of the total herbicide-treated area. During 2005, those lands that could not be treated represented ca. 7.5% of the total area within the herbicide-treated site. During 2006. those non-treatable lands represented 35% of the total herbicide-treated area. Results from this study indicate that managing winter-spring host plants of tarnished plant bug can impact early season tarnished plant bug populations and have implications for management of this pest in adjacent cotton fields. However, the amount of area representing those lands that cannot be treated with herbicides within the crop landscape should be considered before employing this management strategy. The distribution and size of these areas may greatly influence the results and value of this management strategy for tarnished plant bug management in cotton.

# Research article: Sex pheromone of the European tarnished plant bug, Lygus rugulipennis

**DOI:** 10.1673/031.008.4918

**Published:** 2008-09

**Authors:** J.V Cross, P.J Innocenzi, D.R Hall, M.T. Fountain

**Affiliations:** ^1^ East Mailing Research, New Road, East Mailing, Kent ME 19 6BJ, UK; ^2^ Natural Resources Institute, University of Greenwich, Chatham Maritime, Kent ME4 4TB, UK

The European tarnished plant bug, *Lygus rugulipennis*, is an important pest of horticultural crops throughout Europe. *L. rugulipennis* is also an important pest of cucumbers killing growing points and causing misshapen fruits. Adult male *L. rugulipennis* were previously shown to be attracted to traps baited with live virgin females, suggesting that the females produce a sex pheromone. Volatiles produced by virgin female *L. rugulipennis* were shown to contain three components, hexyl butyrate, (E)-2-hexenyl butyrate, and (E)-4-oxo-2-hexenal which elicited electroantennographic (EAG) responses from males in analyses by linked gas chromatography-electroantennography (GC-EAG). They were produced in 1.5:1:0.08 ratio, respectively, by single females and could not be detected in volatiles collected from male *L. rugulipennis*. In field experiments, traps baited with blends of these chemicals dispensed from polyethylene vials and sachets failed to catch significant numbers of males. However, in subsequent field experiments in which the chemicals were released from glass microcapillary tubes, a blend of hexyl butyrate and (E)-4-oxo-2-hexenal was significantly attractive to male *L. rugulipennis*. In addition, while the mixture of all three components attracted fewer *L. rugulipennis* males, this tertiary blend captured significantly greater numbers of males of the congeneric species *L. pratensis* than the binary mixture. The reason why the pheromone blends are attractive when dispensed from glass microcapillaries was not determined. Furthermore, release rates from microcapillary dispensers were erratic; they have only a very short field life and are impractical for operational use. The same three compounds have also been reported to be produced by both females and males of the common green capsid *Lygocoris pabulinus*, an important pest of a wide range of fruit crops in Europe. However, no attraction of males could be demonstrated in the laboratory or field when they were dispensed in various blends and release rates from standard pheromone dispensers. We are embarking on a new 3 year research project to understand the mechanisms and requirements for attraction of three capsid bug species *L*.*rugulipennis, L. pabulinus* and *Liocoris tripustulatus* (a damaging pest of sweet peppers), so that long-lived, practical dispensers can be developed and validated by growers.

# Research article: Distribution & status of Peristenus digoneutis, an introduced parasitoid of Lygus lineolaris in the Northeast U.S.: an update

**DOI:** 10.1673/031.008.4919

**Published:** 2008-09

**Authors:** W.H. Day, K.A. Hoelmer

**Affiliations:** Beneficial Insect Introduction Research Unit, USDA-ARS, Newark, DE, USA

Nymphal parasitism of tarnished plant bugs (*Lygus spp*.) by various species of *Peristenus* (Hym.: Braconidae) in Europe is notably higher (10 to 60 percent) than parasitism of North American *Lygus spp*. by *Peristenus* native to the eastern US (4 to 10 percent). Following a series of earlier releases of European *Peristenus* species elsewhere in North America that failed to result in establishment, a program of releases of *Peristenus digoneutis* obtained from northern Europe was conducted in the NE US from 1979 to 1983 by USDA. Establishment was evident by 1984 at one release site in northern New Jersey. It is now known to be present in 11 northeastern states and the Canadian provinces of Ontario, Quebec and Nova Scotia. *Peristenus digoneutis* has reduced *Lygus* populations in New Jersey alfalfa by 65 percent relative to pre-release levels, and causes moderate to high levels of parasitism in strawberries and apples. However, *P. digoneutis* has not become abundant south of northern NJ (latitude 40.5 degrees N) since its establishment more than 20 years ago. Its southern distribution limit closely matches the summer temperature/duration isocline in this region of 30 degrees C for a maximum of 30 days. The warm summers that are common south of this latitude appear to limit the survival of the *P. digoneutis* population that was established in the NE. Consequently, efforts are underway to establish *P. relictus* (=*P. stygicus*), a related nymphal parasitoid predominant in southern European *Lygus* populations, in the Mid-Atlantic states.

# Research article: Sex pheromones of the green mirid, Creontiades dilutus

**DOI:** 10.1673/031.008.4920

**Published:** 2008-09

**Authors:** Del Socorro, P Alice, T Samuel Lowor, C Peter Gregg

**Affiliations:** ^1^ Cotton Catchment Communities CRC, Narrabri NSW and School of Rural Science & Agriculture, University of New England, Armidale NSW, Australia and; ^2^ Cocoa Research Institute of Ghana, Africa

The green mirid, *Creontiades dilutus* (Stål) (Hemiptera: Miridae) is one of the key species in the “sucking pests complex” in cotton and other field crops such as sunflower, pulses and lucerne in Australia. It is now becoming a significant pest with the reduction of broadspectrum insecticides and the use of more selective chemicals in transgenic cotton. Semiochemicals like sex pheromones might be useful in managing this pest as monitoring tools, in mating disruption or in an attract-and-kill system. An earlier study on the sex pheromones of the green mirid suggested that hexyl hexanoate might be a component, but it was found in both sexes and no males were attracted to this compound (Miles 1995. Identification, pest status, ecology and management of the green mirid, *Creontiades dilutus*, (Stål) (Hemiptera:Miridae), a pest of cotton in Australia. PhD thesis, University of Queensland, Brisbane, Australia). No sex-specific compound was detected. We analyzed air collections and whole body extracts of *C. dilutus* males and females by gas chromatography-mass spectrometry (GC-MS). The sex pheromone was identified as a two-component blend consisting of hexyl hexanoate as the major component and a female-specific compound, (E)-2 hexenyl hexanoate, as the minor component. The latter was detected in the female air collections only. Field trapping experiments were done using Delta traps to compare this two-component blend with either of the single component alone. Only the blend caught males in the traps. Field trials testing various ratios of these components indicated that the optimal ratio was 5:1 of hexyl hexanoate and (E)-2 hexenyl hexanoate. Our preliminary field trials demonstrated that *C. dilutus* pheromones might be useful monitoring tools of mirid populations and in mating disruption. Both uses might be limited by the volatility of the components. A technique we developed to improve the longevity of the pheromone lures in the field is also described.

# Research article: Action thresholds and selective insecticides for management of Lygus in Arizona cotton

**DOI:** 10.1673/031.008.4921

**Published:** 2008-09

**Authors:** P.C Ellsworth, S.E Naranjo

**Affiliations:** ^1^ Arizona Pest Management Center & Department of Entomology, Maricopa Agricultural Center, The University of Arizona, Maricopa, AZ, USA; ^2^ USDA-ARS, Arid-Land Agricultural Research Center, Maricopa, AZ, USA

In most years over the last decade, on a relative basis, cottongrowers have sprayed and spent more to control *Lygus spp*. (*mainly L. hesperus*), and have lost more in yield to this pest than any other insect. As major advances were made in the control of pink bollworm with Bt transgenic cottons and whiteflies [*Bemisia tabaci* (Genn), biotype B] with selective insect growth regulators, *Lygus* bugs have become a more important management focus for growers. Over the last decade, studies in Arizona cotton have focused on the development and refinement of action thresholds, termination rules for chemical control, and the identification and development of selective chemistries that will permit further gains in conservation biological control in our system. We identified 15 total *Lygus* per 100 sweeps as a density associated with economic loss; however, correlations of “total Lygus” with yield were weak. Nymphs, however, especially large nymphs (instars 3–5), were best associated with yield loss over a wide range of conditions. Regression analyses showed maximal yield at 15 total *Lygus* with at least 1.7 nymphs per 100 sweeps. However, revenues were maximized over a wide range of economic conditions at 15 total *Lygus* with at least 5.2 nymphs per 100 sweeps. Extension guidelines were taught to growers and implemented in AZ and Mexico using action thresholds of 15 total *Lygus* with at least 4 nymphs per 100 sweeps (‘15:4’). As the plant senesces and fewer flowering/fruiting sites are produced, the need for and return on control investment diminishes. Termination rules for discontinuing *Lygus* chemical controls over twelve different production scenarios (2 planting dates × 2 irrigation termination timings × 3 different maturity groups) revealed an extremely dynamic relationship between yield/revenue and bug density. Generally, shorter season varieties benefit less from extended protection from *Lygus*. Longer season varieties appear to be especially vulnerable to *Lygus* damage and therefore more responsive to *Lygus* controls. Cotton producers have historically depended on very old and very broad-spectrum chemistry for *Lygus* control, tending to place the grower at greater risk of pest resurgence and secondary pest outbreaks. Exploratory studies with new chemistries over the last 5 years have brought forth two promising control agents, metaflumizone and flonicamid, with new opportunities for selectively controlling *Lygus*. Large plot studies have confirmed that usage of these new compounds leaves a complex of natural enemies intact relative to untreated controls, whereas broad-spectrum standards like acephate dramatically reduce the natural enemy community. The integration of this new understanding in density:damage relationships (e.g., improved action thresholds) and new selective chemistry for control of *Lygus* completes a system for Arizona cotton growers that allows them to manage pests without potentially costly disruptions with broad-spectrum materials while fostering a richer and more functional natural enemy community.

# Research article: Development of species-specific PCR assays for detection and identification of parasitoids associated with Lygus plant bugs

**DOI:** 10.1673/031.008.4922

**Published:** 2008-09

**Authors:** A Martin Erlandson, D Tara Gariepy, Tim Haye, Ulli Kuhlmann

**Affiliations:** ^1^ Agriculutre and Agri-Food Canada, Saskatoon Research Centre, Saskatoon, SK, Canada; ^2^ Dept. Biology, University of Saskatchewan, Saskatoon, SK, Canada; ^3^ CABI Europe-Switzerland, Delémont, Switzerland

*Lygus* plant bugs (Hemiptera: Miridae) are hosts to an array of primary parasitoids which in turn serve as hosts for hyperparasitoids. Members of the genus *Peristenus* (Hymenoptera: Braconidae) are key primary parasitoids in *Lygus spp*. in North America and Europe. In Europe *Peristenus digoneutis* and *P. relitcus* have a significant impact on *Lygus rugulipennis* and have been released in North America in a classical biological control approach against mirid pest species in various crop habitats. The traditional approaches to estimating parasitism in *Lygus spp*. populations include dissection and rearing of field collected nymphs. Although the dissection approach yields relatively quick results, collection of species composition data for the parasitoids is virtually impossible due to the lack of diagnostic morphological characters for *Peristenus spp*. larval stages. Rearing is a time consuming and expensive procedure taking up to 6–9 months to break pupal diapause and for adult eclosion and identification to species based on morphological characters. The rearing approach also suffers from the loss of information due to host and parasitoid mortality during the initial rearing period and overwintering pupal parasitoid mortality. Additionally, the recently described species in the *P. pallipes* complex are difficult for the non-expert to identify even based on adult characters. Thus we have undertaken the development of sensitive and species-specific DNA markers for the detection and identification of key *Peristenus* species associated with *Lygus* pests. A one-step multiplex PCR system based on nuclear rRNA - ITS sequences has been developed and used to detect and distinguish *P*. digoneutis, *P. relictus* and members of the *P. pallipes* complex (Gariepy et al., 2005, Biocontrol Science and Technology 15: 481–495). The multiplex PCR assay was validated in a comprehensive study of parasitism in *L. rugulipennis* populations. Subsequently the multiplex PCR assay has been used in various applications including: ecological host-range testing for *P. digoneutis* and *P. relictus* in their area of origin; detecting the establishment *P. digoneutis* and *P. relictus* in areas of recent releases; and tracking the dispersal of these species from areas of original release. For example, the multiplex PCR system was used to identify *Peristenus* parasitoid species associated with mirid nymphs collected in southern Ontario, including a confirmation of *P. digoneutis* establishment. A number of parasitoid larvae recovered from these mirid nymphs could not be identified using the *Peristenus* multiplex PCR assay; however, the use of universal PCR primers for nuclear rRNA - ITS2 regions and sequencing of the PCR products generated identified these as *Leiophron* uniformis another braconid parasitoid. Further sequence analysis of nuclear rRNA - ITS DNA sequences of geographic populations of the *P. pallipes* complex and other North American *Peristenus* species indicates that the *P. pallipes* complex PCR primer set from the multiplex PCR assay will detect most North American species attacking *Lygus spp*. Finally, to more completely address the potential for species-specific PCR primers to assist in the investigation of host-parasitoid associations, PCR primer sets have been developed for the associated *Mesochorus spp*. hyperparasitoids. Sequence analysis of *Mesochorus spp*. from European mirid populations from different geographic locations and habitats suggest a complex of strains or subspecies of *Mesochorus* attack specific primary parasitoids associated with host mirid species that utilize similar habitats.

# Research article: Economic Impact of Lygus in Arizona Cotton: A Comparative Approach

**DOI:** 10.1673/031.008.4923

**Published:** 2008-09

**Authors:** A Fournier, P.C Ellsworth, V.M. Barkley

**Affiliations:** Arizona Pest Management Center & Department of Entomology, University of Arizona, Maricopa, AZ, USA

In the Western U.S., *Lygus spp.*, especially L. Hesperus, (Hemiptera: Miridae) can cause major losses to cotton, vegetables, seed crops, and a variety of other crops. However, the economic impact of this pest remains largely undocumented in most crops. Two major data sources quantify current management practices and the economic impact of *Lygus* in low-desert upland cotton production in Arizona. A statewide Pesticide Use Reporting (PUR) database, compiled from information reported by applicators to the Arizona Department of Agriculture, documents a high proportion of *Lygus* applications in cotton, though not all types of applications require reporting. In addition, an annual “Cotton Insect Losses” (CIL) survey of cotton Pest Control Advisors (PCAs) helped to provide a more complete picture of statewide *Lygus* management practices. Five years of data (2001–2005) were analyzed. *Lygus* is the most important pest in Arizona cotton most years, based on application*acres of all foliar insecticides. Whitefly [*Bemisia tabaci* (Genn.), biotype B] is the most important *Lygus* co-target, when applications are aimed at controlling more than one pest. The most commonly used foliar materials against *Lygus* in Arizona cotton are acephate, endosulfan and oxamyl, which are typically used at about 90% of maximum label rates. About 80% of *Lygus* applications occur between mid-July and late-August. The two sources of data (CIL & PUR) provide independent estimates of the average number of *Lygus* sprays per acre. Except for 2004, the CIL data estimates somewhat higher insecticide use against *Lygus* than PUR data. Several reasons for this discrepancy were identified, including less than 100% pesticide use reporting in the PUR database; differences in the insecticides included in the estimates; and differences between how the two datasets apportion a single spray event among multiple pest targets. The intensity of *Lygus* insecticide use varies by county. Final County, which has the most cotton acres, shows the highest sprays per acre of the top three active ingredients to control *Lygus*. A section-level analysis of 2005 PUR data indicates a relationship between the intensity of cotton production and spray intensity for *Lygus* control. Growers in Township — Ranges with a low proportion of cotton sections (10–15%) tend to make more sprays on average to control *Lygus*. However, Township — Ranges with the lowest and highest proportions of cotton sections (<10% and >90%) tend to show trends towards fewer sprays for *Lygus* control. These data suggest the possibility that landscape factors can influence *Lygus* populations at the local level, although more research in this area is needed. *Lygus* is perhaps the most significant economic pest of Arizona cotton. Cotton Insect Losses survey data indicate that a high proportion of cotton insect chemical control efforts are directed toward *Lygus* control. About 40% of foliar insecticide sprays target *Lygus*, accounting for about one third of the foliar insecticide budget for growers most years. Despite these control efforts and associated costs, CIL survey respondents consistently rank *Lygus* as the most damaging insect pest of cotton, accounting for more than 50% of insect-related yield loss most years. These data provide important baseline information that will allow us to measure changes in *Lygus* management practices and economic impact over time. Future *Lygus* management practices might be influenced by (1) the introduction of new selective chemistry for *Lygus* control; (2) the introduction of transgenic control options for *Lygus;* or (3) landscape-level changes that could have area-wide impact on *Lygus* management in cotton and other crops. These data underscore the need for continued research to develop effective, selective tools for improved *Lygus* management in cotton, and to integrate these into effective Integrated Pest Management (IPM) programs. There is a need to similarly document the economics of *Lygus* management in other crops including vegetables, seed crops, and alfalfa, and the impact of landscape-level factors on *Lygus* management in a variety of crops.

# Research article: Western tarnished plant bug management in California field crops insecticide efficacy in cotton and dry beans

**DOI:** 10.1673/031.008.4924

**Published:** 2008-09

**Authors:** D Larry Godfrey, W.M Canevari, S.D Wright, T.L Pierce, R.R Lewis

**Affiliations:** ^1^ Dept. of Entomology, Univ. of California, Davis, CA USA; ^2^ Univ. of California Cooperative Extension, San Joaquin County, Stockton, CA USA; ^3^ Univ. of California Cooperative Extension, Tulare County, Tulare, CA USA; ^4^ Dept. of Entomology, Univ. of California Shafter Research and Extension Center, Shafter, CA USA

The western tarnished plant bug, *Lygus hesperus* (Knight) (Heteroptera: Miridae), is a significant pest on several field crops in California. Cotton, both Pima and Acala species, and dry beans, including black-eyed cowpeas, limas, and common beans, can be severely affected. The plant bugs reduce crop yields in cotton and beans as well as impacting crop quality in beans. Biological and cultural methods aid in management of the *Lygus* bugs in field crops, but insecticides are a primary means used to minimize damage. Selected registered and experimental insecticides were compared in terms of efficacy on *L. hesperus*, effects on populations of beneficials, potential to flare secondary arthropod pests, and ability to protect cotton and bean yield and quality in field studies in 2004–2006. A goal was to evaluate the “fit” of the experimental products metaflumizone, flonicamid, and novaluron under California conditions. Among the registered materials, the pyrethroids provided excellent *Lygus* bug control with the organophosphate products giving shortterm control. The pyrethroid insecticides have the drawback of reducing populations of natural enemies and potentially flaring levels of spider mites and aphids.

# Research article: Measuring localized movement of Lygus into cotton

**DOI:** 10.1673/031.008.4925

**Published:** 2008-09

**Authors:** B. Peter Goodell

**Affiliations:** Cooperative Extension, University of California, Statewide IPM Program, Kearney Agricultural Center, Parlier CA

*Lygus hesperus* populations develop both external and internal to the San Joaquin Valley in California. In certain years, weed hosts are favored by precipitation patterns and these can provide extended habitat on which *Lygus* populations can build. In 2005, tarweed, *Hemizonia kelloggii*, was found in high density bordering cotton fields on uncultivated rangeland. *Lygus* populations were sampled weekly from tarweed on uncultivated rangeland and in the adjoining cotton. Both Pima and Acala upland cottons were sampled. In addition to tarweed, almonds (bearing and non-bearing), pistachios, onions and highway frontage were bordering these cotton fields, but were not sampled. *Lygus* population development continued into July on tarweed before soil moisture was depleted and the majority of the tarweed population senesced. *Lygus* population densities did not increase in the cotton most proximal to tarweed until the majority of tarweed in the area had senesced. Based on proximal sampling, other crops that bordered the study area acted as substantial sources for *Lygus* adults during earlier periods of time. These data suggest that *Lygus* remain on hosts as long as possible before finally moving due to unsuitable habitat or habitat destruction caused by production practices. In addition, internal sources (bordering crops) were as significant in providing *Lygus* as was the uncultivated rangeland. This suggests that internal sources of *Lygus* can be as important as external sources and could be more important on an annual basis as opposed to occasional outbreaks from external sources.

# Research article: Status of insecticide resistance and management strategies of Lygus lineolaris in the mid-South

**DOI:** 10.1673/031.008.4926

**Published:** 2008-09

**Authors:** Jeff Gore, Gordon Snodgrass, Angus Catchot

**Affiliations:** ^1^ USDA-ARS, SIMRU, Stoneville, MS, USA; ^2^ Department of Entomology and Plant Pathology, Mississippi State University, Starkville, MS, USA

Multiple experiments were conducted on cotton in Mississippi to investigate management of insecticide resistant tarnished plant bugs, *Lygus lineolaris* (Palisot de Beauvois) (Heteroptera: Miridae). Experiments that controlled native vegetation during the early spring with a single herbicide application reduced tarnished plant bug numbers in cotton (P<0.05) and increased economic returns (P<0.05) for growers in the herbicide treated areas. Another experiment demonstrated the benefits of planting an early season cotton variety (Deltapine 444 BR) versus a later season cotton variety (Deltapine 555 BR). Deltapine 444 BR reached cutout ten days earlier than Deltapine 555 BR and required two fewer foliar applications with insecticides for tarnished plant bugs. Several insecticide screening studies showed that foliar control with currently registered insecticides ranged from 17 to 71 percent control and control with experimental insecticides ranged from 34 to 57 percent control. In an experiment investigating application intervals, plant bug control approached 100 percent when two applications were made four or five days apart and was significantly (P<0.05) better than when the applications were made six (70 percent) and seven (20 percent) days apart, respectively. Rotation with alternative chemistries was shown to improve control of tarnished plant bugs compared to multiple applications with organophosphates. Plots treated with two applications of Orthene (0.5 lb ai/A) five days apart reached threshold significantly (P<0.05) earlier than plots treated with Orthene (0.5 lb ai/A) followed by Centric (2 oz./A). Spray coverage was shown to significantly influence plant bug control. Tarnished plant bug control was significantly higher on okra-leaf cotton than on normal leaf cotton and with hollow cone nozzles than with air induction nozzles. Aldicarb (Temik 15G, Bayer Crop Sciences), applied as a side dress at first flower, reduced plant bug numbers for up to 20 days. Based on these experiments, no one method will provide adequate control of resistant tarnished plant bugs in flowering cotton including foliar insecticides. Therefore, growers and consultants will need to consider alternative strategies integrated with foliar applications to prevent economic losses from tarnished plant bugs.

# Research article: Problems and solutions in the taxonomy of Lygus bugs (Hemiptera: Miridae) parasitoids (Hymenoptera: Braconidae: Euphorinae)

**DOI:** 10.1673/031.008.4927

**Published:** 2008-09

**Authors:** Henri Goulet

**Affiliations:** K. W. Neatby Building, 960 Carling Avenue, Ottawa, ON, Canada

Sixteen species of parasitoids of *Lygus* are known in North America. Four species belong to the genus *Leiophron* and 12 to *Peristenus*. Except for three intentionally introduced species, 13 are native. Most of the species belong to the *Peristenus pallipes* species complex. Classical studies stop here as no character states works. Different approaches were needed to resolve this species complex. Basically, this species complex was resolved by reducing the diversity of species within the complex. One way to reduce the diversity was to look at the complex from a life zone (e.g., cold temperate regions of eastern North America), and an ecosystem within the chosen life zone (e.g., old permanent hay fields sites south of Montreal) followed by weekly analyses of specimens captured at above sites. Synchronous species for a given week sample were generally recognizable. This approach required careful season long field work. Another way to reduce the diversity was to study long series of parasitoids reared from specific hosts (e.g., *Lygus lineolaris* or *L. vanduzei*). This last approach yielded the fastest recognition of cryptic species as only a few species within a species complex may be found within a single host. Rearing is difficult, time consuming and expensive, but the biological data is crucial in species recognition. These parasitoid species are likely to be distinct from one another for a given *Lygus* generation. Long series helped in developing reliable statistics for several characters leading to the discovery of each cryptic species within the species complex.

# Research article: Biology, ecology and management of the green mirid, Creontiades dilutus (Stål) in Australia

**DOI:** 10.1673/031.008.4928

**Published:** 2008-09

**Authors:** C. Peter Gregg

**Affiliations:** Cotton Catchment Communities CRC, Locked Bag 1001, Narrabri, NSW 2390 and School of Rural Science & Agriculture, University of New England, Armidale NSW 2351, Australia

The genus *Lygus* does not occur in Australia. Its nearest analogue, in terms of biology, ecology and pest management, is the green mirid *Creontiades dilutus* (Stål) (GM). Other papers dealing with the damage, economic thresholds, chemical control and pheromones of GM will be presented at this conference. The aim to this paper is to provide an overview of the biology, ecology and management of the species as background to these presentations. *C. dilutus* is one of two significant Australian pest species in the genus *Creontiades Distant*, the other being the brown mirid, *C. pacificus* (Stål). These species attack lucerne, various grain legumes, peanuts and stone fruit, as well as a range of introduced weeds and native host plants. The most important crop affected is cotton, and *C. dilutus* is the most widespread and common of the two species on cotton. Prior to the late 1990's GM was regarded as a minor pest, but with the introduction of single gene transgenic cotton (Ingard®) in 1996, followed by two-gene Bt cotton (Bollgard II®) in 2004, it has become increasingly important. Transgenic Bt varieties now account for about 80% of the Australian cotton crop. Insecticide use against lepidopteran pests, notably *Helicoverpa spp*., has been reduced to about 15% of that on conventional cotton. These insecticides previously reduced numbers of GM, so the species is now emerging as a serious pest, and as an obstacle to IPM in cotton. License costs for transgenic varieties are high, so growers seek to protect their investment and thus economic thresholds for GM are low. Insecticides used against GM are cheap but relatively non-selective, and can flare secondary pests like aphids, mites and whitefly. Knowledge of the biology and ecology of GM is limited, reflecting its recent elevation in pest status. Questions such as whether overwintering reproductive diapause exists, and whether GM is a long-range migrant from inland Australia (as some other cotton pests are) remain unanswered, although some research is in progress. Natural enemies of GM have been identified using ELISA. Spiders, damsel bugs and ladybirds are important generalist predators, but no specific parasites have yet been identified. IPM strategies for GM have been largely limited to the use of lucerne (a highly preferred host) as a trap crop, combined with low rate insecticide/salt mixtures and efforts to promote higher economic thresholds. Current research on biopesticides and semiochemicals may increase the options for IPM. A morphologically similar mirid has recently been reported damaging cotton in southern Texas. It was previously described as *C. dilutus*, but recent molecular, pheromone and morphological evidence indicates that it is probably another, presumably endemic, *Creontiades* species.

# Research article: A molecular approach to quantify predation rates on Lygus hesperus

**DOI:** 10.1673/031.008.4929

**Published:** 2008-09

**Authors:** R. James Hagler

**Affiliations:** Arid Land Agricultural Research Center, USDA-ARS, Maricopa, Arizona, USA

A series of studies were conducted to quantify predation on the western tarnished plant bug, *Lygus hesperus* (Knight) (Heteroptera: Miridae). In a laboratory test I fed predators a single 3rd instar lygus marked with rabbit IgG. The rabbit IgG-marked prey were readily detected in predator guts by an anti-rabbit IgG enzyme-linked immunosorbent assay (ELISA) for up to 24 h after feeding. I then released three uniquely-marked lygus into each of 99 field cages containing the natural arthropod assemblage found on a single cotton plant. Specifically, individual lygus were marked with rabbit IgG, casein, or egg albumin and released into field cages. After 6 h, each caged plant was pulled from the field, the number of predators in each cage was tallied, and each individual predator was assayed for the presence of each protein mark using anti-rabbit IgG, anti-casein, and anti-egg albumin ELISAs, respectively. The average number of naturally occurring predators inhabiting the cages was typically low with < 3.5 predators collected per cage. ELISA results showed that *Hippodamia convergens, Collops vittatus*, and *Nabis alternatus* frequently (e.g., ca. 5 to 10% of the individuals) preyed on marked lygus and only one of the 333 predators examined (*C. vittatus*) scored positive for the presence of two marked lygus in its gut. These studies verified that prey marking is a powerful method for the immunological detection of predation and can be used to study various aspects of predator feeding behavior.

# Research article: Quantifying movement of Lygus hesperus and its natural enemies using a protein mark-capture technique

**DOI:** 10.1673/031.008.4930

**Published:** 2008-09

**Authors:** James Hagler, Jackie Blackmer, Steve Naranjo

**Affiliations:** USDA-ARS, Arid Land Agricultural Research Center, Maricopa, Arizona, USA

A series of tests designed to measure the efficacy of a protein mark-capture technique for the western tarnished plant bug, *Lygus hesperus* were conducted. In laboratory studies, it was determined that *L. hesperus* and *Hippodamia convergens* obtain a protein mark within five minutes after contact exposure to protein-marked cotton leaves. In field cage studies, lygus and potential natural enemies readily acquired and retained a protein mark for several weeks after topical or contact exposure to marked cotton plants. A small scale dispersal study was also conducted to determine if the protein marking procedure was effective at marking *L. hesperus* and *H. convergens*. Specifically, an 18 X 18-m central point section of a 1.4 acre lesquerella field was sprayed with 68 liters of a 10% chicken egg white solution. Sticky traps were then placed at equidistant locations in the lesquerella field, two adjacent 0.9 acre cotton fields, and a 20 acre guayule field. All the lygus and natural enemies (mostly *H. convergens*) trapped after 4 and 7 days were analyzed for the presence of the egg white marker using an anti-egg white enzyme-linked immunosorbent assay (ELISA). A total of 1,067 *L. hesperus* and *H. convergens* were collected from the sticky traps. The overall ELISA results showed that 17% of the individuals contained detectable concentrations of the marker. Of these, 38% were captured in the marked portion of the lesquerella field and 12% were captured in the unmarked portions of the study site. These studies verified that protein marking is a powerful method for studying various aspects of lygus and natural enemy dispersal behavior. This technique will be used in the future to quantify the dispersal characteristics of *L. hesperus* and its natural enemies.

# Research article: Metaflumizone uses in cotton for management of Lygus lineolaris

**DOI:** 10.1673/031.008.4931

**Published:** 2008-09

**Authors:** J.S. Harden, J.B. Guice, L.A. Jose, C.D. Youmans

**Affiliations:** BASF Corporation, Research Triangle Park, North Carolina, USA

Metaflumizone belongs to a new chemical class of insecticides, the semicarbazones, which block the voltage-dependent Na+ channel of susceptible insects (SCBI, IRAC MoA Group 22B) and does not require bio-activation to become an active insecticide. Metaflumizone is active primarily via ingestion. With the advent of Bt containing cotton varieties, there have been shifts in the cotton insect pests from a lepidopteran complex to a *Lygus* (plantbug) complex. The *Lygus* complex has become important across the cotton belt through yield reductions. Laboratory studies indicate that Metaflumizone SC was very active against *Lygus* species. However, early field trails against the two major species of *Lygus, Lygus lineolaris* and *Lygus hesperus*, indicated only moderate control (60 — 70%). Field studies comparing formulations of metaflumizone on *Lygus lineolaris* indicate that foliar treatments of metaflumizone as an EC formulation provides superior control than the SC formulation. Metaflumizone EC at 280 gai/ha was comparable to acephate at 560 — 1120 gai/ha as the standard for *Lygus* control. Mortality was slower to occur than with acephate, but cotton yields were equal. This led to the conclusion that *Lygus* damage to the crop had ceased following the application of metaflumizone. Metaflumizone is being developed as part of a *Lygus* Integrated Pest Management (IPM) program. Treatments which include metaflumizone at 280 g ai/ha are: metaflumizone fb acephate fb dicrotophos, metaflumizone + cyfluthrin fb acephate fb dicrotophos, thiamethoxam fb metaflumizone, acephate fb metaflumizone + cyfluthrin. The standard for comparison is thiamethoxam fb dicrotophos fb acephate. Yields taken from the treatments containing metaflumizone are comparable to that of the standard.

# Research article: Metaflumizone, a new BASF insecticide for Lygus management in cotton

**DOI:** 10.1673/031.008.4932

**Published:** 2008-09

**Authors:** J.S. Harden, J.B. Guice, L.A. Jose, P.M. Munger, T. Sikuljak, C.D. Youmans

**Affiliations:** BASF Corporation, Research Triangle Park, North Carolina, USA

In field studies with *Lygus hesperus*, foliar treatments of metaflumizone at 280 g ai/ha (EC and SC formulations), acephate (1.12 kg ai/ha), or flonicamid (98 g ai/ha) in cotton significantly increased seed cotton yields compared to yields in untreated plots. In order to maintain levels of *L. hesperus* below economic thresholds, four applications of metaflumizone (SC) were required; whereas, only two were needed of metaflumizone (EC), acephate, and flonicamid. Rotational treatment programs of metaflumizone (EC)/acephate, metaflumizone (EC) /flonicamid, and metaflumizone (EC) /flonicamid /acephate resulted in similar outcomes at harvest. Field studies comparing formulations of metaflumizone on *Lygus lineolaris* indicates that foliar treatments of metaflumizone as an EC formulation are superior to that of an SC formulation. Metaflumizone EC at 280 g ai/ha was comparable to acephate at 560 — 1120 g ai/ha as the standard for *Lygus* control. Mortality was slower to occur than with acephate, but cotton yields were equal. This led to the conclusion that *L. lineolaris* damage to the crop had ceased following the application of metaflumizone. Metaflumizone is being developed as part of a *Lygus* Integrated Pest Management (IPM) program. Treatments which include metaflumizone at 280 g ai/ha are: metaflumizone fb acephate fb dicrotophos, metaflumizone + cyfluthrin fb acephate fb dicrotophos, thiamethoxam fb metaflumizone, acephate fb metaflumizone + cyfluthrin. The standard for comparison is thiamethoxam fb dicrotophos fb acephate. Yields taken from the treatments containing metaflumizone are comparable to that of the standard.

# Research article: Flonicamid mechanism-of-action studies using whole-cell patch-clamping of cultured insect neurons

**DOI:** 10.1673/031.008.4933

**Published:** 2008-09

**Authors:** H Jon Hayashi, Geralyn Kelly, P Lyle Kinne

**Affiliations:** ^1^ FMC, Princeton, NJ, USA and Rider University; ^2^ Lawrenceville, NJ, USA

A molecular level understanding of a pesticide's mechanism-of-action is necessary to define the application strategy appropriate to control pests that prey on row crops. Towards this end we have added the standard techniques of whole-cell patch-clamp recording from living insect neurons to our armamentarium in order to resolve the mechanism-of-action of flonicamid, a newly developed pesticidal agent directed against piercing and sucking pests. We found by directly recording ionic current flowing through nicotinic acetylcholine channels (nACh) that flonicamid failed to alter the flow of current through this channel in the three species tested in this work. We conclude, contrary to previous speculation, that flonicamid is not a neonicotinoid. In addition, we have found that flonicamid blockade of the insect A-type potassium channel underlies the lethal effect of this pesticidal agent.

# Research article: Introductions of a European parasitoid, Peristenus relictus (=P. stygicus), against tarnished plant bug in the mid-Atlantic states

**DOI:** 10.1673/031.008.4934

**Published:** 2008-09

**Authors:** Kim Hoelmer, Roger Fuester, Phil Taylor, Dominique Coutinot, Tom Dorsey, Wayne Hudson, George Robbins, Randy Peiffer, Rich Barczewski

**Affiliations:** ^1^ Beneficial Insect Introduction Research Unit, USDA-ARS, Newark, DE, USA; ^2^ European Biological Control Laboratory, USDA-ARS, Montferrier-sur-Lez, France; ^3^ Dept. Alampi Beneficial Insect Laboratory, New Jersey Dept. Agriculture, Trenton, NJ, USA; ^4^ Dept. Agriculture & Natural Resources, Delaware State University, Dover, DE, USA

Populations of tarnished plant bug, *Lygus lineolaris*, in alfalfa have declined by 65% in New Jersey since the establishment in 1984 of *Peristenus digoneutis*, a European nymphal parasitoid of *Lygus* species. Although it has expanded its distribution substantially to the NE and NW, *P. digoneutis* has not become widely established south of its original release site in northern NJ. The recent establishment in California of *Peristenus* from hot-summer regions of Europe suggests that if *P. relictus* (=*P. stygicus* of older literature), the predominant nymphal parasitoid of *Lygus* distributed throughout southern Europe, could be established in the mid-Atlantic states, similar reductions in *Lygus* populations might be obtained. After permission for field release of *P. stygicus* was received, individuals from field collections in eastern and central France (Aveyron & Drome departments) were increased by mass-rearing on a laboratory colony of *L. lineolaris* at the New Jersey Dept. Agriculture Beneficial Insect laboratory. Field releases in New Jersey commenced in 2001. In 2003, the rearing colony was replaced with new stock collected in southern Spain (Andalucia) and central Italy (Umbria), and the host *Lygus* colony was switched to rearing on artificial diet, significantly increasing parasitoid production for field release from 3,200 in 2004 to more than 17,000 in 2005. Beginning in 2006, field releases were made in managed alfalfa fields at 8 sites in New Jersey and Delaware at weekly intervals throughout the summer, totaling ca. 30,000 released. At each site half of the releases were of laboratory-parasitized late-instar nymphs and half were adult parasitoids placed within field cages over alfalfa for one day before cage removal. Releases will be continued in this manner for several years and recovery samples taken to monitor for signs of establishment. Within-season recoveries of progeny were made at two sites in Delaware during 2006. One overwintering female *P. relictus* was also recovered in 2006 from a 2004 release site in New Jersey planted with a wildflower mixture.

# Research article: Effects of flonicamid (Carbine) on Lygus hesperus feeding behavior

**DOI:** 10.1673/031.008.4935

**Published:** 2008-09

**Authors:** P. Houston Joost

**Affiliations:** FMC Corporation, Princeton, NJ, USA

Flonicamid is a novel chemistry which causes permanent feeding cessation in some Homopteran and Heteropteran pests. In this study we used DC electrical penetration graph technique (EPG) to determine how quickly flonicamid affects the feeding behavior of *Lygus hesperus. Lygus hesperus* were placed on either an untreated greenbean or a greenbean treated with flonicamid at a rate of 0.099 ai kg/ha. Probing duration, probes with ingestion frequency, ingestion duration and percent of ingestion per probe of *L. hesperus* on the flonicamid treated greenbeans were all significantly reduced in a 6 hr trial. *Lygus hesperus* on flonicamid treated greenbeans ingestion duration was reduced by 50% (2189s to 1110s) in the first hour after exposure and reduced by 99% (2390s to 166s) in the second hour of the trial compared to *L .hesperus* on untreated greenbeans. Results indicate that *L. hesperus* feeding is inhibited within an hour of exposure to flonicamid.

# Research article: Damage assessment and action threshold for mirids, Creontiades spp. in Bollgard II cotton in Australia

**DOI:** 10.1673/031.008.4936

**Published:** 2008-09

**Authors:** M Khan, A Quade, D Murray

**Affiliations:** ^1^ QDPI&F, Plant Science, Kingaroy, QLD, Australia; ^2^ QDPI&F, Plant Science, Toowoomba, QLD, Australia

Two species of mirids, green mirid (GM), *Creontiades dilutus* (Stal) and brown mirid (BM), *Creontiades pacificus* (Stal), have emerged as important sucking pests in Bollgard II cotton in Australia. In conventional cotton mirids have been well studied as pests of the seedling and squaring stage, while in Bollgard II cotton they are found throughout the season and little is known about their damage in the boll stage. We conducted a series of glasshouse and field trials to understand mirid damage in Bollgard II cotton and to develop an action threshold for the pest. Trials showed that while GMs were abundant throughout the season, BM's were found mainly later in the season and GMs were the dominant species. The nature of damage for both GM and BM at boll stage was similar. At boll stage mirid damage caused black spots on the boll wall, warty growth inside the boll wall and brown colored lint. Bolls up to 20 days old incurred significantly more damage then older bolls; preferred age being 15 days or less and bolls less than 7 days old could abort. All stages of mirids caused damage to bolls and damage is cumulative. However, 4th and 5th instar nymphs and adults caused significantly more damage than younger nymphs. Trials also showed that in Bollgard II cotton at squaring stage (from emergence to 60% plants with first flower), plants recovered later in the season from the damage caused by 4 mirids per meter provided fruit loss was <40%. Early boll stage (from 60% plants with first bolls to 60% bolls at 25 days old) was the critical stage for mirid damage and 3 to 4 mirids per meter caused significant yield loss. At late boll stage (from 60% boll at 25 days old to harvest) mirids failed to cause any significant yield loss. These findings were used to recommend and action threshold for mirids in Bollgard II cotton in Australia.

# Research article: Reduced rate of chemical plus additive - an effective IPM tool for managing mirids, Creontiades spp. in Australian cotton

**DOI:** 10.1673/031.008.4937

**Published:** 2008-09

**Authors:** M Khan, A Quade, D Murray

**Affiliations:** ^1^ QDPI&F, Plant Science, Kinagroy, QLD, Australia; ^2^ QDPI&F, Plant Science, Toowoomba, QLD, Australia

In Australia, problems with mirids, Creontiades spp. have increased substantially following the adoption of Bollgard II cotton and 2 to 4 insecticide sprays were required in different valleys to manage them. Unfortunately in Australia most of the registered chemicals for mirids are non-selective and are highly disruptive to a wide range of beneficial species. Use of these chemicals in early or mid season will undermine IPM approaches and potentially flare other pests such as mites, whitefly and aphids later in the season. We conducted trials using an additive, salt (NaCl), with reduced rates of fipronil (Regent) and indoxacarb (Steward) to find out if mixtures can be used as an IPM tool for managing mirids. Trials showed that salt with reduced rate insecticide increased mortality by 20 to 30 percent compared to low rate insecticide alone without reducing residual effectiveness compared with the full rates. Trials also showed that the impact of salt mixture on beneficial species was reduced substantially compared to full rate of chemicals.

# Research article: Insecticide use strategies in mid-south cotton fields for tarnished plant bug

**DOI:** 10.1673/031.008.4938

**Published:** 2008-09

**Authors:** Roger Leonard, Don Cook

**Affiliations:** ^1^ Louisiana State University AgCenter, Macon Ridge Research Station, Winnsboro, LA, USA, USA; ^2^ Mississippi Agricultural and Forestry Experiment Station, Verona, MS, USA

The tarnished plant bug, *Lygus lineolaris* (Palisot de Beauvois), is a common pest of cotton throughout the southern and southeastern areas of the U. S. Cotton Belt. Yield losses in cotton due to this pest vary temporally and spatially. The primary factors for these variations in population levels and economic injury include: (1) environmental conditions favorable for tarnished plant bug population growth and development, (2) prolonged weather events that result in preferred wild hosts becoming less attractive, (3) environmental conditions that stress cotton plants and results in greater sensitivity to tarnished plant bug injury, and (4) efficacy of insecticides registered for tarnished plant bug control. Across the mid-south states of Arkansas, Louisiana, and Mississippi during 1991–2005, tarnished plant bug has infested 77–99 percent of cotton acreage. During those years, 44–93 percent of the infested acres were treated with one or more insecticide applications. The frequency of insecticide applications has varied during the past 15 years, but a general increase has occurred across the entire period. During 1991–1993, the annual number of insecticide applications targeting tarnished plant bugs was less than one. During 2003–2005, producers applied 2.3–4.4 insecticide treatments per year for this pest. The costs of these control strategies have increased 10-fold, from $5 million to greater than $50 million during the previous 15 years. In spite of inputs for insecticide use strategies, estimates of the average cotton yield losses attributed to this pest during the five year period of 2001–2005 are higher than that observed for any year in recent history. Historically, the tarnished plant bug has been considered an early season pest of cotton and has been of little concern after the initiation of flowering. The cotton plant is most susceptible to yield losses from tarnished plant bug injury between the four to six true leaf stages through early squaring. Tarnished plant bug injury generally results from feeding on small flower buds (squares), which abscise from the cotton plant. Tarnished plant bug feeding on bolls was considered to be a relatively non-significant source of yield loss. However, this insect has become a primary pest of cotton plants during the flowering and boll maturation periods. This pest can induce abscission until bolls accumulate greater than or equal to 245 heat units after anthesis. Seedcotton yields may be significantly reduced by tarnished plant bugs until bolls accumulate at least 330 heat units after anthesis. Given the opportunity, tarnished plant bugs are capable of significant injury to cotton during the flowering period. Historically during the flowering and boll maturation periods, tarnished plant bugs were inadvertently controlled by organochlorine, organophosphate, carbamate, and pyrethroid insecticides targeting boll weevil, *Anthonomus grandis grandis* (Boheman) and heliothines. The success of the boll weevil eradication programs and wide-scale adoption of *Bacillus thuringiensis* Berliner var. *kurstaki* cotton cultivars eliminated many of those applications. In addition, the use of more selective insecticides that target Lepidopteran pests (i.e. spinosad, indoxacarb, and emamectin benzoate) have increased survival of tarnished plant bugs. Finally, tarnished plant bug resistance to a variety of insecticides (organophosphates, carbamates, pyrethroids) in the mid-south states has contributed to the elevated status of this insect pest. The action thresholds for tarnished plant bug in pre-flowering cotton have been well-established for several years, and recent research efforts are generating data to further support those recommendations. However, with considerable changes in cotton production, IPM practices, and the pest status of tarnished plant bug during the last decade, the action thresholds in flowering cotton are a topic of considerable research interest. The current recommendations are mostly qualitative and based on experience of the pest manager rather than quantitative scientific data. This problem is compounded by the difficulty in sampling for tarnished plant bugs in flowering cotton. In general, sweep nets are the preferred method for sampling tarnished plant bugs in pre-flowering cotton. However, sweep net samples do not provide an accurate estimate of tarnished plant bug population densities during the flowering period of cotton plant development. The most accurate method for estimating tarnished plant bug densities in flowering cotton appears to be the drop cloth. Drop cloth samples provide an adequate method for estimating populations, but are considered to be time efficient for practical application by agricultural consultants and farm managers. Federal and state entomologists in the mid-south have several ongoing projects to refine sampling protocols and action thresholds for tarnished plant bug. A series of trials compared and calibrated all recommended sampling methods including visual observation, drop cloth, and sweep net based upon the present action thresholds. Another study validated flower bud (square) injury as an effective measurement of insect damage. The results indicate that an actual treatment threshold to manage this pest should be in the range of 10–20 percent injured squares. A third group of experiments is examining a series of action thresholds during the flowering and boll maturation stages under a range of environmental conditions throughout the region. Satisfactory control of tarnished plant bug in mid-south (Arkansas, Louisiana, Mississippi) cotton fields with insecticides has become difficult in recent years, especially during the mid-to-late season. Insecticides recommended for tarnished plant bug include compounds in the organophosphate, carbamate, neonicotinoid, pyridine carboxamide, and IGR chemical classes. Of all those listed, acephate and dicrotophos are the most frequent compounds selected by producers due to their concurrent efficacy against other Heteropteran pests of cotton. Mid-south populations of tarnished plant bug have been shown to express varying levels of resistance to several of these insecticides and instances of unsatisfactory control have become common. The agrochemical industries are aggressively pursuing research and development of novel insecticides, but there are limited alternatives to currently recommended products. To prolong the efficacy of available products, insecticide use strategies recommended by mid-south entomologists are based upon temporal restrictions and a rotating schedule. During the pre-bloom stages of cotton development, the organophosphate insecticides are not recommended, and if tarnished plant bug populations exceed action levels for treatment, other products should be applied. During the flowering and boll maturation stages, organophosphates are the primary insecticides. Producers are discouraged from using subsequent applications of the same product and should rotate with compounds in other classes before returning to an organophosphate treatment. The IGR, novaluron (Diamond), also is recommended in co-applications with other products to improve residual efficacy against immature stages of tarnished plant bugs. The tarnished plant bug has become the new key pest of cotton in the mid-south region of the U.S. Considerable research efforts are underway to validate and improve sampling protocols and action thresholds during the early-, mid-, and late-seasons of cotton development. Many of the recommended insecticides are not providing consistent control; and novel compounds are desperately needed. The evolving insecticide use strategies that are in place promote careful selection of products for use during specific periods of plant development.

# Research article: Managing Lygus in seed alfalfa

**DOI:** 10.1673/031.008.4939

**Published:** 2008-09

**Authors:** C Shannon Mueller, B Peter Goodell

**Affiliations:** ^1^ University of California Cooperative Extension, Fresno, CA, USA, Statewide IPM Program, Kearney Agricultural Center, Parlier, CA, USA

In the San Joaquin Valley of California, *Lygus* bugs (*Lygus spp*.) are a pest throughout the season, shifting between crops within the landscape as plants become unsuitable hosts due to maturity or harvest. *Lygus* are the key insect pest and by far the most difficult insect to manage in alfalfa seed fields, and when present in high numbers, may completely destroy the crop. Both adults and nymphs feed on the alfalfa plant, attacking reproductive parts, and causing premature drop of buds and flowers (stripping), seed deformation, and reduced seed viability. Control of *Lygus* is critical to the economic production of alfalfa seed. Action thresholds vary with the stage of crop development. The greatest period of *Lygus* bug activity is from June through August. Degree-days can be used to forecast seasonal *Lygus* development, especially hatch and migration dates. Pesticide applications should be timed to coincide with the hatching of *Lygus* broods. Treatment can be delayed until egg hatch is complete, but should take place before the nymphs reach the fourth and fifth instar since these older instars and adults are more difficult to control with insecticides than younger instars. Few insecticides are available for the control of *Lygus* in alfalfa seed fields. Because options are limited, and there is a high potential for the development of resistance, it is critical to maintain and preserve the efficacy of currently registered chemicals. Key components in the ability of *Lygus* to develop resistance to insecticides include short life cycle with many generations per year, a wide host range, and exposure to many insecticide applications each year, not only in seed alfalfa but in other susceptible crops as well. The best insurance against development of insecticide resistance is rotating chemical controls and maintaining the insect's natural enemies in the field. Research continues to develop alfalfa germplasm with resistance to *Lygus*.

# Research article: A new insecticide for management of Lygus Hesperus in western U.S. crops

**DOI:** 10.1673/031.008.4940

**Published:** 2008-09

**Authors:** P.H Munger, T Sikuljak, L Jose, J.S. Harden

**Affiliations:** BASF Corporation, Research Triangle Park, NC, USA

Metaflumizone is a new active ingredient under development by BASF, which in research studies has demonstrated significant activity against important Lepidoptera, Coleoptera, Hemiptera, Hymenoptera, Isoptera, and Diptera. Metaflumizone belongs to a new chemical class of insecticides, the semicarbazones, which block the voltage-dependent Na+ channel of susceptible insects (SCBI, IRAC MoA Group 22B). Metabolic bio-activation of metaflumizone is not a pre-requisite for insecticidal activity. Metaflumizone is active primarily via ingestion. However, in laboratory studies mortality of *Lygus hesperus* was also observed when insects were treated posttopically with metaflumizone. In those studies, solutions prepared using an EC formulation of metaflumizone were more efficacious at 24 hours after treatment than those prepared using an SC formulation. In field studies with *Lygus hesperus*, foliar treatments of metaflumizone at 280 g ai/ha (EC and SC formulations), acephate (1.12 kg ai/ha), or flonicamid (98 g ai/ha) in cotton significantly increased seed cotton yields compared to yields in untreated plots. In order to maintain levels of *L. hesperus* below economic thresholds, four applications of metaflumizone (SC) were required; whereas, only two were needed of metaflumizone (EC), acephate, and flonicamid. Rotational treatment programs of metaflumizone (EC) /acephate, metaflumizone (EC) /flonicamid, and metaflumizone (EC) /flonicamid /acephate resulted in similar outcomes at harvest. In field trials on dry beans, treatments of metaflumizone (240 or 280 g ai/ha) and lambda- cyhalothrin (38 g ai/ha) significantly decreased seed damage, increased seed quality and dry bean yields compared to the untreated plots. Lambda-cyhalothrin was generally more effective than metaflumizone in reducing numbers of *L. hesperus* nymphs. In cotton, *L. hesperus* captured from metaflumizone-treated plots are alive, but not actively feeding /damaging the crop.

# Research article: Survey and evaluation of Lygus bugs on lesquerella and guayule, two new desert crops in the western USA

**DOI:** 10.1673/031.008.4941

**Published:** 2008-09

**Authors:** S.E Naranjo, D Dierig, P.C Ellsworth

**Affiliations:** ^1^ USDA-ARS, Arid-Land Agricultural Research Center, Maricopa, AZ, USA; ^2^ Dept. of Entomology, University of Arizona, Maricopa Agricultural Center, Maricopa, AZ, USA

*Lesquerella spp*. (Family Brassicaceae) are perennials native to North America and their seeds contain industrially-valuable hydroxy fatty acids used for the production of bio-lubricants, motor oils, resins, waxes, nylons, plastics, corrosion inhibitors, cosmetics and coatings. *Lesquerella fendleri* occurs in the southwestern USA and northern Mexico and research to develop this species as an annual alternative crop for the arid southwest has been underway since the mid 1980s. Guayule, *Parthenium argentatum* (Family Asteraceae), is a perennial shrub native to the Chihuahuan desert of North America and is a source of hypoallergenic latex useful in making all types of rubber products. Guayule has been used as a source of natural rubber at various times over the past 100 years in the USA and renewed interest in the crop emerged in the past decade when better methods for extracting the latex were developed. It is now grown commercially in Arizona. Nothing is known about the arthropods inhabiting either of these new crops. Survey studies initiated in *L. fendleri* in 2004 reveal that it hosts large populations of *Lygus spp*. (primarily *L. hesperus*) along with a high diversity and abundance of other arthropods, many of which are beneficial species shared with cotton, alfalfa and other crops in Arizona. In 2005, experimental studies manipulating *Lygus* bug densities with insecticides showed that increasing densities of *Lygus* bugs were significantly related to increasing levels of fruit abortion, and flower bud and seed pod damage. This overall damage resulted in significant decreases in yield and marginally significant reductions in seed oil content. Similar studies in 2006 showed trends of increasing levels of fruit abortion, and flower bud and seed pod damage with increasing densities of *Lygus* bugs, but no effects were observed on yield. A final year of study is underway in 2007. Year-round survey studies in commercial guayule were initiated in 2005. Many of the same arthropods found in lesquerella also are present in guayule but at much lower densities. *Lygus* bugs are present over an extended portion of the year but generally absent during winter months. Natural enemy populations are present year-round. Additional work is planned to examine the pest potential of *Lygus* bugs in this crop. Further studies are also examining the potential role of both lesquerella and guayule as sources of natural enemies for other crops such as cotton.

# Research article: Lygus spp. as pests of cotton in the United States

**DOI:** 10.1673/031.008.4942

**Published:** 2008-09

**Authors:** F. Patricia O'Leary

**Affiliations:** Cotton Incorporated, Cary, NC, USA

*Lygus spp*. are important pests of cotton throughout much of the U.S. cotton belt. This presentation provides an overview of the current status of the pests and research being conducted to meet the challenges of an evolving pest complex and production system.

# Research article: Effects of seeding date, canola species and pesticides on Lygus bugs in Alberta, Canada

**DOI:** 10.1673/031.008.4943

**Published:** 2008-09

**Authors:** J Otani, H Cárcamo, L Dosdall, R Blackshaw, G Clayton, N Harker, K Turkington, J O'Donovan

**Affiliations:** ^1^ Beaverlodge Research Farm, Agriculture and Agri-Food Canada, Beaverlodge, Alberta, Canada; ^2^ Lethbridge Research Centre, Agriculture and Agri-Food Canada, Lethbridge, Alberta, Canada; ^3^ Department of Agricultural, Food and Nutritional Science, University of Alberta, Edmonton, Alberta, Canada; ^4^ Lacombe Research Centre, Agriculture and Agri-Food Canada, Lacombe, Alberta, Canada

Plant bugs of the genus *Lygus* (*L. lineolaris, L borealis, L. keltoni*) are common in canola fields throughout Alberta, although, the species complex varies in each region, and require insecticide spraying at the pod stage in localized areas in some years. We conducted field studies from 2001 to 2004 at Lethbridge (south), Lacombe (central) and Beaverlodge (north) to assess the impacts of seeding date (fall, April and May), and canola species (Polish = *Brassica rapa* cv., Argentine = *B. napus* cv. Q2) as main plot factors with pesticide spraying (control, fungicide, insecticide, fungicide plus insecticide) as a split factor. *Lygus* bug numbers were low at all sites most years. However, in at least one of the 3 years at each site *Lygus* bug populations surpassed the economic threshold of 2 *Lygus* bugs per sweep. At Beaverlodge plots seeded in April tended to have higher *Lygus* bugs than those planted in May but at Lacombe in 2002 and Lethbridge in 2004, the opposite trend was observed where plots seeded in May had higher *Lygus* bug infestations than those seeded earlier in April. In general, *B. rapa* tended to attract slightly higher numbers of *Lygus* bugs than *B. napus*. Yields generally increased with increasing pesticide inputs but detailed economic analyses remain to be done. Correlations examining the relationship between *Lygus* adult or nymph densities with yield (kg/ha), thousand-seed weights (gm), seed protein (%) or oil content of seed (%) for *B. rapa* cv. Hysynl 10 and *B. napus* cv. Q2 were performed at three target stages in the above years. The three target stages of “before insecticide”, “after insecticide”, and “mid-pod stage” depended on crop stage as opposed to calendar date between years and sites and were intended to validate the current economic threshold for *Lygus* bugs in canola which recommends use of insecticide at the early pod stage. No significant correlations were observed between *Lygus* bug abundance and seed yield at Lethbridge. *Lygus* bug populations were relatively low at Beaverlodge throughout the duration of the study reaching seasonal maximums of 3.2 bugs per sweep in 2001, 5.2 bugs per sweep in 2002, 3.4 bugs per sweep in 2003, and 3.4 bugs per sweep in 2004, whereas at Lacombe seasonal maximums of 28.0 bugs per sweep in 2002 and 22.5 bugs per sweep in 2004 were observed. *Lygus* bug densities failed to show a significant relationship with thousand-seed weights and seed protein at Beaverlodge and Lacombe. There were significant, negative relationships between *Lygus* bug populations and yield and oil content of canola seed and most arose at Lacombe where higher densities of *Lygus* bugs were observed in plots that enjoyed better seasonal growing conditions. More specifically, data for Beaverlodge and Lacombe was separated into canola species and correlations were examined between *Lygus* adult or nymph densities and the above harvest variables. Incidents of significant, negative Pearson's correlation coefficients occurred at Lacombe in 2002 in comparisons of *Lygus* adult densities collected “before insecticide” and yield of Hysyn 110 (r = -0.73, p<0.0001, N=47) but also between adult densities collected “after insecticide” and yield of Hysyn 110 (r = -0.59, p<0.0001, N=47). Significant, negative correlations occurred at Lacombe in 2002 between adult densities collected “before insecticide” and yield in Q2 (r = -0.56, p<0.0001, N=44) but also between adult densities “after insecticide” and yield (r = -0.68, p<0.0001, N=48). Significant, negative correlations similarly occurred at Lacombe in 2004 between adult densities collected “mid-pod” and yield (r = -0.59, p<0.0001, N=48). These results suggest a trend wherein the longer-season *B. napus* cv. Q2 suffered more than the shorter-season *B. rapa* cv. Hysyn 110 when *Lygus* populations were high and canola had good growing conditions. Interestingly *Lygus* nymph densities failed to show significant correlations in these comparisons with yield, thousand-seed weights, seed protein or oil content of seed. Correlations examining *Lygus* populations in fall, April or May seeding dates with the above harvest parameters revealed very few significant relationships. There was no relationship between *Lygus* numbers thousand-seed weights, seed protein, or oil content of seed at Beaverlodge or at Lacombe. There were three incidents of a significant relationship between adult densities and yield and these all occurred at Lacombe in 2002. There was a significant, negative relationship between *Lygus* adult populations occurring at “mid-pod” and yield at Lacombe in 2002 (r = -0.58, p<0.0001, N=31). Additionally, there was a significant, negative relationship between nymph densities at “mid-pod” and yield at Lacombe in 2002 in both the fall (r = -0.61, p<0.0005, N=32) and April seeding dates (r = -0.76, p<0.0001, N=31). *Lygus* bugs were not the primary insect pest in canola plots grown in southern Alberta yet results from central and northern Alberta suggest a trend wherein canola species, rather than a specific canola seeding date, was affected to a greater degree by naturally occurring infestations of *Lygus* bugs. These results suggest that the longer-season *B. napus* cv. Q2 may suffer more from natural *Lygus* bug infestations than the shorter-season *B. rapa* cv. Hysyn110 when grown in the central or northern canola growing regions of Alberta.

# Research article: Efficacy against Lygus rugulipennis of insecticides commonly used in IPM peach orchards of NW Italy

**DOI:** 10.1673/031.008.4944

**Published:** 2008-09

**Authors:** Marco Pansa, Luciana Tavella

**Affiliations:** Di.Va.P.R.A. Entomologia e Zoologia applicate all'Ambiente “Carlo Vidano”, University of Torino, Italy

Since the 1990s, severe injuries to fruits due to bug feeding have been reported in several peach orchards of NW Italy. Investigations carried out in 1992–1994 showed that the species responsible for damage was the European tarnished plant bug, *Lygus rugulipennis* Poppius (Heteroptera: Miridae), and that an accurate weed management in the orchards, i.e. mowing of alternate rows, prevented the plant bugs from migrating to peach trees (Tavella et al. 1997. IOBC/WPRS Bulletin 20 (6): 1–5). However, severe damage to peach has been recently reported also in well-managed orchards, so research has been carried out in 2004–2006 to evaluate the efficacy against *L. rugulipennis* of some insecticides commonly used in orchards: spinosad, thiacloprid were evaluated in laboratory (Snodgrass GL. 1996. Journal of Economic Entomology 89: 783–790) and semifield trials. In the laboratory, etofenprox and malathion determined a high mortality, whereas spinosad and thiacloprid did not show any efficacy against *L. rugulipennis*. In semifield trials, mortality rates were lower than those assessed in laboratory trials, and variable in the 3 replications. Anyway, the efficacy of etofenprox and malathion was strongly reduced when the insects were introduced into the cages one week after the treatment. Overall, in both laboratory and semifield conditions the most effective a.i. against *L. rugulipennis* was malathion, which reduced significantly populations of other plant bugs in trials carried out on birdsfoot trefoil in North America (Wipfli MS et al. 1990. Journal of Economic Entomology 83: 2086–2091). By contrast, etofenprox showed different toxicity levels in relation to the commercial formulation, so further investigations are required to evaluate its actual efficacy in controlling plant bug infestations.

# Research article: Intercrop movement of Lygus hesperus in the Texas High Plains: Potential for landscape level management

**DOI:** 10.1673/031.008.4945

**Published:** 2008-09

**Authors:** N Megha Parajulee, B Ram Shrestha, C. Stanley Carroll

**Affiliations:** Texas Agricultural Experiment Station, 1102 East FM 1294, Lubbock, TX 79403

*Lygus hesperus* (Knight) (Hemiptera: Miridae) is an economically important pest of various field crops. It is the dominant species within the Texas High Plains *Lygus* complex. The year-round availability of its potentially preferred host plants is one of the reasons for the occurrence of this pest throughout most of the year in the Texas High Plains. Because it is a highly polyphagous and mobile pest, it can exploit various field crops and weed hosts for its population development and maintenance. It has been recorded that *L. hesperus* can move from one host plant to another alternative host plant species when the primary host becomes unsuitable or less preferred. Thus, management of this pest is not feasible by simply managing a single crop, which leads to the need for landscape level management of this pest. Intensive surveys of all potential field crops and wild weed hosts of *Lygus hesperus* have been conducted for the Texas High Plains region which provides a listing of potential hosts, host-plant sequencing and indirect assessment of intercrop movement of this pest. Because alfalfa is a major host plant of *L. hesperus* in the Texas High Plains cotton production landscape, a two-year season long survey of roadside alfalfa and adjacent cotton was conducted at numerous locations in Lubbock County. A two-year field study was also conducted in selected weed and crop hosts to examine *Lygus* colonization preference. These two indirect assessments of *Lygus* movement suggested selected weed and crop hosts to examine *Lygus* colonization preference. These two indirect assessments of *Lygus* movement suggested alfalfa with cotton may largely determine the resulting intercrop movement of *Lygus* between cotton and non-cotton hosts. Therefore, if alfalfa is not managed properly it may serve as a source of *Lygus* for adjacent cotton. Physical marking and recapture studies have been reported for some other crops but the technique of physical marking with fluorescent dye is not efficient and cannot be used for field marking and landscape level monitoring of a *Lygus* population. The enzyme-linked immunosorbent assay (ELISA) has been successfully used for other insects for quantification of their intercrop movement. Therefore, we tested the ELISA technique to quantify cotton-alfalfa intercrop movement of *L. hesperus*. Seven animal origin proteins and one plant protein were tested for their suitability in ELISA based *Lygus* monitoring. The direct ELISA protocol has been optimized for two animal proteins for laboratory and field marking. Chicken egg albumin and bovine milk casein can be used for field marking in landscape level monitoring of intercrop movement of *L. hesperus*. A preliminary study conducted in 2006 under a low *Lygus* density regime showed that ELISA detection procedure satisfactorily quantified the inter- and intra-crop movement between alfalfa and cotton. ELISA detected that 1 out of 3 *Lygus* retrieved from cotton moved from adjacent alfalfa while 4 out of 85 *Lygus* retrieved from alfalfa showed positive response to markers from both alfalfa and cotton, indicating likely movement from alfalfa to cotton and back to alfalfa. More research is underway to examine the *Lygus* movement behavior at the landscape level.

# Research article: Morphometry of Lygus bugs: Implications in pest management

**DOI:** 10.1673/031.008.4946

**Published:** 2008-09

**Authors:** N Megha Parajulee, B. Ram Shrestha

**Affiliations:** Texas Agricultural Experiment Station, 1102 East FM 1294, Lubbock, TX 79403

The genus *Lygus* (Hemiptera: Miridae) consists of 43 described species worldwide. Since there is no published information on the molecular phylogenetic and biological species determination for *Lygus*, the most common way of species determination is based on a phenotypic species concept using taxonomic keys. Other species concepts are difficult and not practicable in field level species identification; therefore phenotypic species identification is the common practice and will remain popular in the future. Taxonomic keys that have been developed use very few phenotypic characters of *Lygus* and some characters are largely qualitative and very difficult to quantify. Thus, special taxonomic skills in related taxa are necessary for better identification at the species level. Moreover, taxonomic keys cannot be used to identify cryptic species or hybridization between species. Due to difficulty in species identification and occurrence of multiple species of *Lygus* in most ecosystems, many scientists had combined all species and reported them as a “*Lygus* complex.” Because there exists a considerable variation among species in terms of their population dynamics, damage potential, host preference, and insecticide resistance, lack of proper species identification limits the ability to develop more effective *Lygus* pest management strategies. A comprehensive morphometric phylogenetic study at the *Lygus* species level is a reasonable solution for this problem until we can develop molecular phylogenetic techniques for this genus. Morphometric studies were conducted on *Lygus* species collected from alfalfa fields in the Texas High Plains region. Morphometric measurements of various body parts located on the dorsal side of adult *Lygus* were taken using digital image analysis while the computations were performed using MATLAB functions. Various geometric parameters including surface area, length, shape, roundness, and convexity of six body parts were measured and analyzed using PCA, one-way ANOVA and stepwise discriminate analysis. The detail protocol of morphometry using digital image analysis and comparison between traditional morphometry and landmark based morphometry will be discussed. Clavus convex hull perimeter, dorsal body length, clavus area, dorsal body perimeter, and clavus length were the most informative characters among the 36 measured variables showing higher loading values for the major principal components. These characters were similar in both male and female specimens. Morphometric comparison between *Lygus hesperus* and *Lygus lineolaris* was done by using digital image analysis of 12 morphometric characters. The *Lygus hesperus* specimens had significantly larger pronotum, longer rostrum and longer second antennal segment as compared with that of *Lygus lineolaris*.

# Research article: Measuring genetic variation of tarnished plant bug, Lygus lineolaris (Palisot de Beauvois), over temporal and spatial scales

**DOI:** 10.1673/031.008.4947

**Published:** 2008-09

**Authors:** P Omaththage Perera, Jeff Gore, Gordon Snodgrass, Craig Abel

**Affiliations:** Southern Insect Management Research Unit, USDA-ARS, Stoneville, MS 38776, USA

Five *Lygus lineolaris* populations were collected over a six month period along approximately 10 mile transect in Washington County, MS. Collections made in May, July, and September, 2006 were analyzed using 14 polymorphic microsatellite loci. Allele frequencies were used to estimate the Nei's genetic diversity parameters. The total genetic diversity (Ht), the within population genetic diversity (Hs), the among population genetic diversity (Dst) and the coefficient of genetic differentiation (Gst) estimates were 0.485, 0.453, 0.032, and 0.066, respectively. Overall gene flow estimates, calculated using private allele method, between populations in May, July, and September were 2.66, 12.96, and 16.43, respectively. The data suggest minimal genetic differentiation among populations in the study area and a correlation between gene flow and increase in population size.

# Research article: Colonization of Lygus nymphal parasitoids in the Monterey Bay region

**DOI:** 10.1673/031.008.4948

**Published:** 2008-09

**Authors:** Pickett Charles, Dagne Duguma, Michelle Lawson, Sean Swezey, Janet Bryer, Diego Nieto, Martin Erlandson, Mark Bolda, Ramana Colfer

**Affiliations:** ^1^ Biological Control Program, California Department of Food and Agriculture, CA, USA; ^2^ Department of Entomology, University of Arkansas, Fayetteville, AR, USA; ^3^ Ecology and Sustainable Systems, University of California, Santa Cruz, CA, USA; ^4^ Agriculture and Agri-Food Canada, Saskatoon, Saskatchewan, Canada; ^5^ UC Cooperative Extension, Watsonville, CA, USA; ^6^ Mission Organics, San Juan Bautista, CA, USA

The nymphal parasitoid *Peristenus digoneutis* Loan (Hymenoptera: Braconidae) was successfully imported into eastern United States during the 1980's for control of *Lygus lineolaris* (Palisot de Beauvois) infesting alfalfa. The resulting control of *L. lineolaris* renewed interest in doing the same for control of its close relative in California, *Lygus hesperus* Knight. *Peristenus digoneutis* has recently been reported attacking *L. lineolaris* infesting strawberries in New York. We have successfully imported and colonized both *P. digoneutis* and *Peristenus relictus* (Ruhte), formerly *P. stygicus* Loan in Sacramento, California. Beginning in 2002 we began releasing the same parasitoids at locations in the Monterey Bay region. Today, there are 4 release sites in this region: two of natural vegetation bordering or near strawberry production, and another two on organic strawberry farming systems. *Peristenus relictus* has been recovered 2 years after being last released into the initial study site of natural vegetation. It has been recovered one year since last released into strips of alfalfa interspersed in strawberry fields and used as a trap crop for *Lygus spp*.

# Research article: Management of plant bugs in Europe under greenhouse condition

**DOI:** 10.1673/031.008.4949

**Published:** 2008-09

**Authors:** Birgitta Rämert, Barbro Nedstam, Erika Qvarfordt, Karolina Åsman

**Affiliations:** ^1^ Department of Plant Protection Biology, The Swedish University of Agricultural Sciences (SLU), Alnarp; ^2^ Swedish Board of Agriculture (SJV), Alnarp; ^3^ Swedish University of Agricultural Sciences (SLU), Swedish Species Information Centre, Uppsala

In Sweden infestation of plant bugs *Lygus spp*. and *Liocoris tripustulatus* (Fabricius) (Heteroptera: Miridae), the common nettle capsid bug, in greenhouse production of cucumber is an increasing problem. Feeding damage on young shoots may cause plant deformations and developing fruits may become unmarketable. Today there is no selective insecticide available for use against mirids in cucumber production in Sweden. However, the use of bio insecticides, such as insect pathogenic fungi may be a promising alternative to conventional chemicals. To develop a management program of mirids in greenhouse production, pilot studies were undertaken. During February – September 2004 a survey was carried out in several greenhouses to find out which species dominated and how early they occurred and if any species could overwinter in the greenhouses. Yellow and blue sticky traps (Catch-it) were placed in half of the greenhouses from the week after planting until the end of March to see if the traps could be used as indicators of plant bug infestations. During spring and early summer, mirids was also surveyed on herbaceous vegetation surrounding the greenhouses. Thereafter, the susceptibility of one of the *Lygus* species *Lygus pratensis* (L.) (Heteroptera: Miridae) towards the commercially bioinsecticide BotaniGard ES (Beauveria bassiana strain GHA) was evaluated both in a laboratory and in a greenhouse study. In all greenhouses *Lygus rugulipennis* (Poppius) (Heteroptera: Miridae), the European tarnished plant bug, and *L. tripustulatus*, were found, with L. *rugulipennis* as the dominating species. The same two species were also the most common ones on the vegetation surrounding the greenhouses, with *L. tripustulatus* dominating on nettles. In some of the greenhouses adult bugs were found already in February and feeding damage was obvious on plants within two weeks after planting. The sticky traps were not useful as indicators for adults overwintering in greenhouses. Symptoms of bug activities were already developing on the plants when the first specimen of *L. tripustulatus* was trapped. The pathogenicity of BotaniGard ES was tested at different concentrations on hibernated *L. pratensis* sampled in May and on adults sampled from the summer generation in August to calculate LC 50 values. The BotaniGard ES was pathogenic to adults from both the hibernated and summer generation. The LC50 value for total mortality of *L. pratensis* 8 days after treatment was higher for the hibernated generation compared to the summer generation, indicating that the summer generation is more susceptible. In the greenhouse experiments BotaniGard ES was applied at two different dosages (250 and 125 ml/L) on *Lygus spp*. nymphs feeding on cucumber plants. An application with only water was also made as a control. At termination 8 days after treatment the total mortality of the nymphs was significantly higher on the nymphs treated with the higher dose compared to the nymphs feeding on the control plants (just water application) but the mortality was the same between the control and the lower dose treatment. To be able to discover and remove the overwintering and migrating insects early in the season and during replanting in July further studies are needed to find suitable and efficient traps or trap crops. More efficient application techniques using BotaniGard for both adults and nymphs is in need of development. The suppression of mirids using different formulations and doses of BotaniGard is in need of refinement.

# Research article: Molecular phylogeny and genetic diversity of Lygus

**DOI:** 10.1673/031.008.4950

**Published:** 2008-09

**Authors:** Richard Roehrdanz, Robert Foottit, Prasad Burange, Eric Maw, Mark Boetel, Sheila Sears

**Affiliations:** ^1^ Insect Genetics & Biochemistry, Red River Valley Agricultural Research Center, ARS, USDA, Fargo, ND, USA; ^2^ Canadian National Collection of Insects, AAFC, Ottawa, ON, Canada; ^3^ Department of Entomology, North Dakota State University, Fargo, ND, USA

The genus *Lygus* (Heteroptera: Miridae) in North America includes several species of polyphagous insect pests. The most common of these in the eastern half of the USA and Canada is the tarnished plant bug, *Lygus lineolaris*, which has been observed feeding on over 300 different plants. In western North America it is replaced primarily by the western tarnished plant bug, *L. hesperus*, and to a lesser extent *L. elisus, L. keltoni*, and *L. borealis*. There are additional *Lygus* species that are not agricultural pests. Inter- and intraspecific genetic diversity in North American *Lygus* was examined using nuclear and mitochondrial DNA. DNA sequences have been obtained from the mitochondrial cox1 and cox2 genes, the nuclear ITS1 spacer, and some microsatellite flanking regions (MSFR). The Fargo lab sequenced a region overlapping the cox1-cox2 boundary, whereas the Ottawa lab sequenced a region near the 5-prime end of cox1. These data sets are being merged by reciprocal swapping and sequencing of key DNA samples. *L. lineolaris* has been widely sampled in the eastern US and southern Canada. The cox1-cox2 boundary sequence identified two major clades and a small third group of very divergent individuals. One of the MSFRs and cox1 together also form two clades. The three marker sets appear to be defining the same populations. Both clades are widely distributed and sympatric. Clade 1 is most common in southern Ontario and eastern North Dakota. Clade 2 predominates in the rest of Canada from Quebec to Alberta and also represents the majority of insects from New England and the Gulf Coast in the USA. The data raises the possibility of cryptic species within *L. lineolaris*. Except for the paucity of Clade 1 at most Canadian sites, well-defined geographic based population structure has not been uncovered in *L. lineolaris*. The molecular and morphological data both support the monophyly of the genus *Lygus* with *HenryLygus* as the sister taxon. At the interspecific level within the genus both ITS1 and MSFRs roughly divide the species into eastern and western groups. *L. borealis* is eastern with ITS1 and western with MSFRs. Strict monophyly of the species has been difficult to demonstrate. With cox1-cox2 the majority of sequences from individuals identified as *L. lineolaris, L. elisus, L. borealis, L. hesperus*, and *L. keltoni* cluster as species. However a few individuals from all of these species are scattered among other branches of the trees. Morphological species assignment is difficult because of overlapping characters and the misplaced individuals are most likely the result of these difficulties. The *L. hesperus* and *L. keltoni* clusters are intermixed in both cox1-cox2 and some MSFR. Further evaluation of their species status is in progress. The complete mt genome (∼ 14,150 bp), except for the control region and some sequences adjacent to the control region has been sequenced from *L. lineolaris*. Stephen Cameron (Canberra, Australia) has sequenced the same region from *L*. hesperus. The nucleotide sequence divergence between them is about 3.4% and the gene order of both corresponds to that for *Drosophila yakuba*.

# Research article: Examining the dispersal capabilities of Lygus hesperus and its natural enemy Geocoris pallens in California's San Joaquin Valley

**DOI:** 10.1673/031.008.4951

**Published:** 2008-09

**Authors:** J Frances Sheller, A Jay Rosenheim, R James Hagler

**Affiliations:** ^1^ Department of Entomology, University of California, Davis, CA, USA; ^2^ USDA-ARS, Arid-Land Agricultural Research Center, Maricopa, AZ, USA

In order to manage the western tarnished plant bug (*Lygus hesperus* Knight) from a landscape perspective, it is essential to understand its dispersal capability. In this preliminary study we measured the dispersal of *L*. hesperus and its natural enemies from a cut alfalfa field into adjacent cotton fields. *L*. hesperus will leave a host field when it becomes unsuitable, usually when it is harvested or senesces. The monthly cutting of alfalfa for hay commonly creates such a scenario. In this experiment we marked a 72-acre alfalfa field that had a substantial density of *L*. hesperus (6–7 *Lygus* per 100 sweeps) with a distinctive protein mark (a diluted cow's milk solution). The following day the alfalfa field was cut, presumably forcing its occupants to disperse. Cotton fields adjacent to the eastern border of the cut alfalfa field were sampled the day immediately after cutting and again 6 days later. In addition to *Lygus spp.*, predators including *Geocoris spp., Orius insidiosus, Nabis spp., Hippodamia convergens*, and *Coccinella septempunctata* were collected, with the majority of samples collected being *Geocoris pallens*. Understanding how these predators disperse may increase our ability to utilize them as effective biological control agents. Samples were then analyzed with enzyme-linked immunosorbent assay (ELISA) to detect the protein mark (3093 individuals). This analysis detected marked *L. hesperus* up to 800 meters into the cotton field. This preliminary result suggests that some small fraction of a *L. hesperus* population disperses strikingly long distances and that long-distance dispersal may be an important factor in the colonization of California cotton fields.

# Research article: The role of diapause in the remarkable adaptation of the tarnished plant bug to its environment in the mid-South

**DOI:** 10.1673/031.008.4952

**Published:** 2008-09

**Authors:** Gordon Snodgrass

**Affiliations:** USDA-ARS, Stoneville, MS, USA

Tarnished plant bugs, *Lygus lineolaris* (Palisot de Beauvois), overwinter as diapausing adults throughout North America. Because of the importance of diapause in the development of control methods for plant bugs, diapause in the tarnished plant bug was studied from 1999–2007 in field and laboratory tests at Stoneville, MS. Stoneville is at a latitude of 33.43 N and is in Washington County near the western edge of MS in the Mississippi River Delta. Tarnished plant bugs begin entering diapause at Stoneville in late August and by 12 September about 50% of the developing nymphs will produce diapausing adults. Most nymphs developing in the field at the shorter day lengths found in October and November become diapausing adults. However, reproduction in the fall continues in the field on weed hosts until the hosts are killed by a freeze or senescence occurs. Reproduction can also occur during the winter with new generation adults produced in March in mild winters. Tarnished plant bugs are able to break diapause and utilize favorable weather conditions to reproduce in the fall and winter in the mid-South because of two factors. One factor is the presence of abundant wild hosts that bloom in October and November along with winter hosts (mainly henbit, *Lamium amplexicaule L*.) that bloom in late November through March. The other factor is the ability of diapausing adult tarnished plant bugs to break diapause under a diapausing-inducing day length in response to temperature and food stimuli. Both of these stimuli are important, and food must contain nutrients as are found in blooms or flower buds. The laboratory and field tests that determined the importance of food and temperature in the emergence of plant bugs from diapause and how this makes plant bugs so well adapted to their environment in the mid-South are discussed.

# Research article: Control of western tarnished plant bug Lygus hesperus Knight (Heteroptera: Miridae) in California organic strawberries using alfalfa trap crops and tractor-mounted vacuums

**DOI:** 10.1673/031.008.4953

**Published:** 2008-09

**Authors:** L Sean Swezey, J Diego Nieto, A. Janet Bryer

**Affiliations:** Center for Agroecology and Sustainable Food Systems, University of California, Santa Cruz, 95064

A key economic pest of strawberries in California is the western tarnished plant bug, *Lygus hesperus* Knight (Heteroptera: Miridae). Alfalfa (*Medicago sativa* L.) is a highly attractive plant host to western tarnished plant bug, and we hypothesized that it can be successfully managed as a trap crop for pest suppression in strawberries. Completely randomized design trap cropping experiments were established on an organic strawberry farm from 2002–2004. Western tarnished plant bug adults and nymphs were significantly more abundant in alfalfa trap crops than in comparable edge strawberry rows. Over three experimental years, twice-weekly summer vacuuming of alfalfa trap crops with a tractor- mounted vacuuming device reduced adult and nymph abundance by 72 and 90% respectively in trap crops. This summer vacuuming of alfalfa trap crops also significantly reduced damage due to western tarnished plant bug in associated un-vacuumed organic strawberries June and July 2002, June 2003 and June and July 2004), compared with either an untreated control (2003) or the organic strawberry growers' standard whole-field vacuuming treatment. Vacuuming of alfalfa trap crops reduces an organic grower's costs (tractor, tractor fuel, and driver time) by 78% when compared with current whole field vacuuming practices. An economic analysis of a whole ha model indicates that a positive return from the use of vacuumed trap crops could be realized in 2004. The overall potential positive net return for the three months of vacuumed alfalfa trap crop treatments in 2004 was calculated at +$ 1,829 per ha.

# Research article: Population dynamics of Lygus rugulipennis in agroecosystems of NW Italy

**DOI:** 10.1673/031.008.4954

**Published:** 2008-09

**Authors:** Luciana Tavella, Marco Pansa

**Affiliations:** Di.Va.P.R.A. Entomologia e Zoologia applicate all'Ambiente “Carlo Vidano”, University of Torino, Italy

In NW Italy severe damage due to the European tarnished plant bug *Lygus rugulipennis* Poppius (Heteroptera: Miridae) has been reported above all on peach and strawberry. So research was carried out in 2004–2006 to assess seasonal abundance and movement of *L. rugulipennis* inside and outside crops. In peach orchards, the plant bug generally lives and reproduces on herbaceous plants, and migrates onto peach trees when winter cereals are harvested and usual host plants are lacking. Overall, in the investigated orchards, damage to peach fruits collected was rare until mid-July when adults migrated into fields from other host plants such as alfalfa and clover; in this period the plant bug could cause serious injuries to fruits.

# Research article: Techniques for Evaluating Feeding Preferences of Lygus lineolaris in Midsouth Cotton

**DOI:** 10.1673/031.008.4955

**Published:** 2008-09

**Authors:** Tina Teague Gray, Jennifer Lund, J Twinkle Sangepogu, M Freddie Bourland

**Affiliations:** ^1^ Arkansas State University, University of Arkansas Agricultural Experiment Station, Jonesboro, AR, USA; ^2^ University of Arkansas Northeast Research and Extension Center, Keiser, AR, USA

Field and laboratory studies to develop and improve methodology for screening promising cotton lines with host plant resistance to tarnished plant bug (*Lygus lineolaris*) were initiated in this Cotton Incorporated sponsored project in Arkansas in 2005. Field evaluation methods included measures of tarnished plant bug population densities, cotton square retention, and anther damage in squares and flowers. Results from standard field observations in small plot tests were compared with results from lab preference studies made using a laboratory feeding bioassay under development. In these initial studies, evaluations were made with cotton germplasm lines with distinct morphological traits including lines with and without extrafloral nectaries, with normal and Frego bracts, and lines with normal and early fruiting characteristics. Extrafloral nectaries provide feeding sites in pre-flower cotton for insects. Frego bract cotton types have bracts that are rolled and twisted leaving the floral bud (square) and fruit (boll) exposed. The early fruiting lines can have a lower first fruiting node compared to other lines. Earlier availability of squares can have a lower first fruiting node compared to other lines. Earlier availability of squares could affect adult *Lygus* feeding preferences in early season. A major goal in these first 2 years has been to develop laboratory screening techniques that could have practical utility in cotton breeding programs. One approach used small testing arenas where plant bug nymphs were presented squares from different cotton lines including small and large squares, and squares with and without bracts. Disposable plastic food containers lined with moistened florist water foam (bottom area of 100 cm2) and with the top fitted with netting were used for all tests. Three field collected nymphs (3 or 4th instar) were released in test containers with squares arranged on the water foam in either 2×2, 3×3 or 4×4 latin square designs. Nymphs were removed after 24 hrs; squares were held an additional 24 hrs and then dissected to evaluate anther damage. Nymphs preferred the Frego cotton type in the laboratory tests compared to two commercially available cultivars and a nectariless line from the breeding program. Differences in presence of bracts or square size were not significant factors in plant bug feeding preference. Results from laboratory assessments were comparable to measurements made for tarnished plant bug preferences in the field. The water foam arena test shows promise as a simple laboratory technique that allows rapid assessment of plant bug feeding preferences. Additional work will be needed to explore the utility of this technique to support on-going cotton breeding efforts in the Midsouth.

# Research article: Challenges of managing Lygus in strawberry plantations on the central coast of California

**DOI:** 10.1673/031.008.4956

**Published:** 2008-09

**Authors:** G. Frank Zalom

**Affiliations:** Department of Entomology, University of California, Davis, CA, USA

*Lygus* bugs are a serious pest of strawberries grown in California's Central Coast area and Santa Maria Valley where fruit are produced through the summer months. *Lygus* are rarely pests in southern California and the Central Valley where harvest is generally completed by the end of June. However, *Lygus* damage may occur on second-year plantings and when berry plants remain in the field through the Summer and into Fall. The main species present is *Lygus hesperus*, but a second species, *Lygus elisus*, can also be found in mixed populations. Adult *Lygus* bugs typically overwinter from September to January on flowering plants and legumes outside of strawberry plantations. Cover crops including clover, fava beans and vetch are good *Lygus* hosts. Some *Lygus* over-winter on summer planted strawberries which become infested during the Fall migration and on second-year strawberries which had become infested during the first year. Three seasonal populations of nymphs occur on central coast strawberries, the first in May or early June, the second in late June or early July, and the third in late July or August, depending on location and temperature conditions. Adults from the third generation overwinter, and will lay eggs in the Spring, contributing to the next season's problem. Feeding by the second hatch of nymphs on strawberries usually causes the greatest damage if not controlled, however monitoring and management of all nymphal hatches is important. *Lygus* nymphs and adults feed on strawberry seeds and both are capable of damaging fruit. When a seed is damaged, the flesh behind it does not develop properly creating a characteristic discoloration and malformation called “cat-facing”. Damaged strawberries will not recover, so it is necessary to suppress *Lygus* before the damage occurs. Successful *Lygus* management requires an understanding of its biology and life cycle, and is best achieved through a combination of control approaches and careful monitoring both within strawberry plantations and on its weed hosts. A *Lygus* phenology model has been adapted from the cotton safflower system of California's San Joaquin Valley which can be used to predict when *Lygus* nymphs will be present in weeds and in strawberries. Controlling *Lygus* nymphs is preferred because they can't migrate from weed hosts or trap crops that are destroyed when they are present, and nymphs are also more susceptible to control using insecticides. Treatment thresholds for *Lygus* in strawberries is very low because of the value of the strawberry crop. The gross return for a hectare of California strawberries approaches $125,000. Treatment is recommended when 1 *Lygus* is found in 10 plants when sampling by suction machine, or at 1 *Lygus* per 20 plants when sampling by beating tray. Therefore, insecticides are widely used for controlling *Lygus*, but there are few registered products that are effective. Pesticide registrations are difficult to obtain on strawberries because of the relatively limited area on which they are planted (about 10,500 hectares), the frequency of harvest, and frequent worker entry into the plantations. Those products that are registered must therefore be used wisely to maintain their efficacy. Vegetation management in areas nearby strawberry plantations is important to reduce local *Lygus* populations that can serve as sources of infestation. Weed abatement and trap crops are both employed by some growers, but there are challenges in implementing both approaches to prevent these alternate *Lygus* hosts from instead becoming sources of strawberry damage. Tractor mounted suction machines, or “bug vacs” are also used by some growers, but their efficiency can vary considerably depending on machine.
